# Electric Field Susceptibility of Chlorophyll c Leads to Unexpected Excitation Dynamics in the Major Light-Harvesting Complex of Diatoms

**DOI:** 10.1021/acs.jpclett.3c03241

**Published:** 2024-02-27

**Authors:** Sayan Maity, Vangelis Daskalakis, Thomas L. C. Jansen, Ulrich Kleinekathöfer

**Affiliations:** † School of Science, Constructor University, Campus Ring 1, 28759 Bremen, Germany; ‡ Department of Chemical Engineering, School of Engineering, University of Patras, Patras 26504, Greece; ¶ Zernike Institute for Advanced Materials, University of Groningen, Nijenborgh 4, 9747 AG Groningen, Netherlands

## Abstract

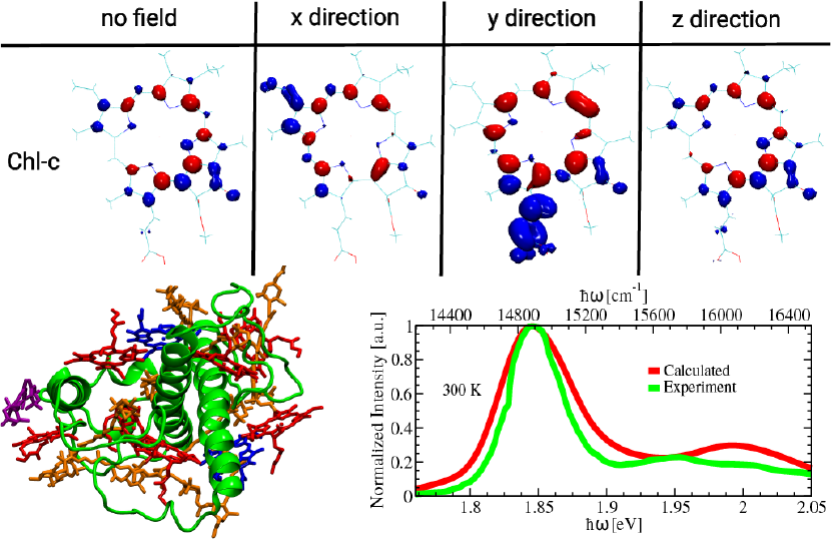

Diatoms are one of the most abundant photosynthetic organisms on earth and contribute largely to atmospheric oxygen production. They contain fucoxanthin and chlorophyll-a/c binding proteins (FCPs) as light-harvesting complexes with a remarkable adaptation to the fluctuating light on ocean surfaces. To understand the basis of the photosynthetic process in diatoms, the excitation energy funneling within FCPs must be probed. A state-of-the-art multiscale analysis within a quantum mechanics/molecular mechanics framework has been employed. To this end, the chlorophyll (Chl) excitation energies within the FCP complex from the diatom *Phaeodactylum tricornutum* have been determined. The Chl-c excitation energies were found to be 5-fold more susceptible to electric fields than those of Chl-a pigments and thus are significantly lower in FCP than in organic solvents. This finding challenges the general belief that the excitation energy of Chl-c is always higher than that of Chl-a in FCP proteins and reveals that Chl-c molecules are much more sensitive to electric fields within protein scaffolds than in Chl-a pigments. The analysis of the linear absorption spectrum and the two-dimensional electronic spectra of the FCP complex strongly supports these findings and allows us to study the excitation transfer within the FCP complex.

Diatoms are marine algae that produce about 40–50% of the marine biomass with a 20–25% contribution to global primary production via photosynthesis for the entire food web on earth. They also contribute to atmospheric carbon fixation.^1−3^ Like higher plants, the light-harvesting (LH) process within diatoms is performed by protein-pigment complexes embedded within thylakoid membranes. The major LH complex of diatoms is a fucoxanthin and chlorophyll binding protein (FCP) containing fucoxanthin carotenoids in place of the xanthophylls present in higher plants.^4^ Although the LH complexes of higher plants and diatoms belong to similar protein classes with comparable sequences, their absorption differs substantially due to the presence of different pigment types.^5^ The large amount of fucoxanthin carotenoids contained in the FCP complexes enables the absorption of sunlight in the blue-green region, which allows diatoms to perform photosynthesis in an aquatic environment.^6^ Furthermore, the presence of chlorophyll-c (Chl-c) molecules in the diatom FCP complexes, as opposed to the Chl-b present in higher plants, adds toward this goal.

The first crystallographic structure of an FCP complex belongs to the diatom *Phaeodactylum tricornutum* and shows a dimeric unit^7^ whereas later studies on the organism *Chaetoceros gracilis* found different tetrameric as well as monomeric units near the PSII core of those diatoms based on cryo-electron microscopy.^8,9^ In addition, for the same complex a PSI-FCP complex has been resolved.^10^ Furthermore, a structure for the PSII-FCPII supercomplex from the organism *Thalassiosira pseudonana* was recently reported.^11^ Although the polypeptide structures of these FCP complexes are quite similar, they differ in the chlorophyll (Chl) and carotenoid content. The monomeric FCP unit from *P. tricornutum* contains nine chlorophyll molecules (seven Chl-a and two Chl-c molecules), seven fucoxanthin (Fx) molecules, and one diadinoxanthin (Ddx) molecule. In the case of the diatom *C. gracilis*, the PSII core is surrounded by two homotetramers and three dissimilar FCP monomers. The homotetramers are named MT (“moderately” bound tetrameter) and ST (“strongly” bound tetrameter). Each monomer unit of these tetramers (FCP-A) contains seven Chl-a, three Chl-c, and seven Fx molecules. The monomeric units around PSII are called FCP-D, FCP-E, and FCP-F. The FCP-D monomer is found at the interface between the MT-tetrameter and the CP43 complex of the PSII core. This FCP-D monomer binds nine Chl-a, one Chl-c, and six Fx pigment molecules. Moreover, the structural location of the FCP-D monomer is analogous to that of the CP29 minor antenna on the periphery of the PSII complex of higher plants. The FCP-F contains seven Chl-a, two Chl-c, and six Fx molecules, whereas FCP-E binds two additional Chl-a molecules and one additional Fx molecule. Moreover, the last two monomeric units contain one Ddx carotenoid molecule each. Interestingly, none of the FCP monomeric units from the organism *C. gracilis* shows the same Chl/carotenoid ratio as that of the structurally resolved FCP protein of the diatom *P. tricornutum*. At the same time, their stoichiometry does not match the FCP complex from *Cyclotella meneghiniana* whose structure has been resolved^12^ recently, but based on experimental studies, it was predicted to contain eight Chl-a, two Chl-c, and eight Fx molecules.^13,14^ More details of the structural differences between the FCP complexes of the two diatom organisms *P. tricornutum* and *C. gracilis* are given in ref (5). Moreover, a structural model of the trimeric unit of the FCP complex from *C. meneghiniana* has been predicted in ref (5) based on two-dimensional spectroscopy^15−17^ which needs to be compared with a recently solved structure that contains eight Chl-a, three Chl-c, and seven Fx carotenoid molecules per monomer in a trimeric unit.^12^

The large number of carotenoid molecules within FCP complexes suggests their crucial role in the photosynthetic process of the diatoms or its regulation on the turbulent ocean surface. The down-regulatory mechanisms of photosynthesis in diatoms are triggered by an enhanced proton gradient across the thylakoid membrane.^18,19^ Moreover, the presence of LHCX class proteins and xanthophyll-cycle carotenoids^20,21^ can potentially lead to an aggregation of FCP complexes^22,23^ that regulates their transition between light-harvesting and photoprotective states (down-regulation of photosynthesis). Based on classical molecular dynamics (MD) simulations, we have recently identified a remarkable flexibility in the FCP scaffold of the diatom *P. tricornutum* with the Chl-a 409/Fx-301 pigment pair therein to become a possible key player in the transition between different states of the FCP complex.^24^ This was later confirmed by experimental spectroscopy.^25^ However, a key question remains: What is the advantage of embedding Chl-c within the FCP pigment network of diatoms, and what is the effect of a flexible protein matrix on that network?

The present study focuses on the diatom *P. tricornutum* and the impact of the FCP protein matrix on the excitation energy distributions of the different Chl molecules therein. The lowest excitation energies, i.e., the *Q*_*y*_ excitation energies, are often also termed site energies in so-called tight-binding models of the Chl network. A structural overview of the FCP complex from *P. tricornutum* is shown in Figure 1 along with its Chl network. Only very recently, an exciton Hamiltonian for this system was determined, but with the stark difference that the excitation energies were determined without taking the protein environment into account.^26^ As will be shown in this study, the effects of the protein environment on the excitation energies are, however, surprisingly large in the FCP complex and cannot be neglected. Another study looked at the shift in excitation energies upon changing the protonation state of the acrylate group in Chl-c using a QM/MM scheme.^27^ The focus of that study was rather narrow on this specific topic. None of these studies looked at the Q_*y*_ excitation energies for all pigments in the FCP complex in a QM/MM fashion, in comparison to the associated energy funnel. In vacuum and in organic solvents, the Q_*y*_ excitation energies of the Chl-c molecules are known to be blue-shifted compared to those of the Chl-a molecules.^28^ Hence, at first sight, a fast energy transfer is expected from the Chl-c to the Chl-a pigments inside the FCP protein. This assumption agrees with earlier experimental studies where an extremely fast energy transfer from the Chl-c to the Chl-a molecules was reported to have been observed in pump–probe and two-dimensional spectroscopy experiments for the FCP complex of the diatom *C. meneghiniana* at low temperature and at room temperature.^13,15,17,29^ In these studies, the Q_*y*_ excitation energies of Chl-c molecules were about 0.1 eV higher than those of Chl-a molecules.^17^ For such an energetic arrangement, energy funneling can happen from the Chl-c to the Chl-a pigments on an ultrafast time scale. Moreover, a similar interpretation was given for the experimental findings of the FCP complex from the *C. gracilis* organism.^30,31^

**Figure 1:**
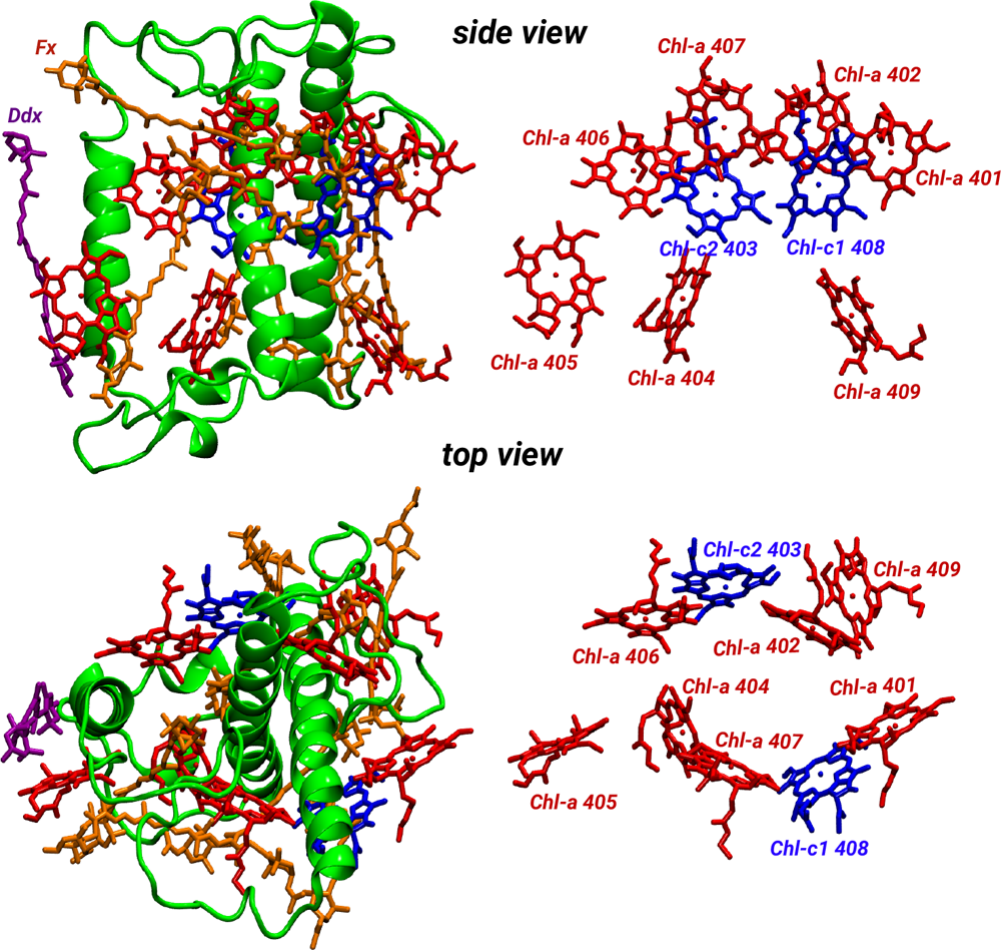
Structure of the FCP complex from *P. tricornutum* depicted in a green cartoon representation. The Chl-a and Chl-c molecules are shown in red and blue, respectively, while the carotenoids Fx and Ddx are presented in orange and violet, respectively. In addition, the arrangement of the Chl molecules is delineated in the right panel together with the respective labels according to the crystal structure^7^ (pdb code: 6A2W). The top and bottom panels represent side and top views of the same complex, respectively.

In the present study, we go beyond the current experimental and theoretical knowledge and probe the energetic position of each pigment molecule and thus the energy funnel within the FCP complex from *P. tricornutum*, for which a crystal structure is available. For this purpose, we have performed a multiscale simulation with the numerically efficient density-functional-based tight-binding (DFTB) approach at its center.^32^ Surprisingly and counterintuitively, our simulations show that the Chl-c pigments have average excitation energies lower than or at most similar to that of any other Chl-a molecule in the FCP complex from *P. tricornutum*. This result also takes into consideration thermal fluctuations via a 1 ns-long quantum mechanics/molecular mechanics (QM/MM) MD simulation employing the time-dependent long-range-corrected (TD-LC) DFTB method within the QM/MM scheme. The energetic order of the average excitation energies along the QM/MM MD trajectory stayed almost the same as for the QM/MM-minimized and crystal structure conformations. These results suggest that the energy transfer direction within the FCP complex from *P. tricornutum* is different from what was proposed earlier for another FCP complex^16,17^ and that the Chl-c2 pigment might be acting as a terminal emitter in the energy funnel under certain conditions. This also differs from the experimental findings for the FCP complex from another diatom, i.e., *C. meneghiniana*,^13,15−17,29^ and questions the assigned energy flow pathways in various diatom organisms. In addition to the excitation energies, we have calculated the linear absorption spectrum of the FCP complex and compared it to the experimental literature.^7,33^ Only a minimal contribution of the Chl-c molecules to the total absorption was observed, whereas the Chl-a pigments made the largest contribution. Interestingly, the high-frequency sideband of the FCP absorption spectrum is solely attributed to the vibrational sideband of the Chl-a molecules. This finding strongly questions earlier assumptions that the high-frequency sideband in the linear absorption spectrum of the FCP complex from the diatom *C. meneghiniana* is due to the major contribution of Chl-c molecules only.^16,17^ To support this claim, we performed additional calculations using the numerical integration of the Schrödinger equation (NISE) method^34−36^ to model two-dimensional electronic spectra (2DES) of the FCP complex. The results of these calculations can be directly compared with the experimental findings for the FCP complex from the diatom *C. meneghiniana*. Our analysis of the 2DES spectra unveiled a rapid decay of the vibrational peaks, predominantly originating from Chl-a pigments. Within a mere 50 fs, the intensity of these peaks decays to just 20% of their initial value. This swift decay aligns nicely with experimental findings but had previously been assigned solely to the transfer of excitons from Chl-c to Chl-a.^13,16,17^ However, during this previous assignment no structures of FCP complexes were available, and studies like the present one were not yet possible. Additionally, employing the NISE method, we determined the population transfer dynamics, observing an overall relaxation of the population between the Chl-a and Chl-c chromophores occurring on a picosecond time scale.

The letter is organized as follows: first, the excitation energy ladder of the FCP complex is reported using various quantum chemical methods based on the QM/MM-optimized structure as well as along a trajectory. Subsequently, we discuss the differences between the electronic ground and excited state densities that are determined to identify the electrostatic effects of the protein environment on the *Q*_*y*_ absorption of individual Chl molecules. Then, the electric field susceptibility of Chl-a and Chl-c pigments is reported. Furthermore, we computed the linear absorption spectrum of the FCP complex, highlighting the contributions from the Chl-a and Chl-c molecules. Finally, we establish a direct connection to experimental measurements by analyzing 2DES. At the end of the letter, the physiological consequences of the findings are discussed in relation to the experimental literature and especially regarding the potential implications on the exciton dynamics of the studied FCP complex.

After the missing heavy and hydrogen atoms were added, the FCP structure from *P. tricornutum* (pdb: 6A2W) was considered to be a rigid model in a first step. Then, a QM/MM optimization was performed on an equilibrated FCP model that included the thylakoid membrane and aqueous phases as designed at neutral pH in our previous study.^24^ We have determined the Q_*y*_ excitation energies for the individual Chl molecules based on the crystal structure conformation and the QM/MM-optimized geometry. Figure 2A depicts the Q_*y*_ excitation energies based on the QM-MM optimized geometry which form the energy ladder in the FCP complex of the diatom *P. tricornutum* calculated at different levels of theory (details in Materials and Methods in the Supporting Information). The DFT/MRCI calculations are computationally more demanding than the LC-DFT formalism and have been shown to produce quite accurate excitation energies for Chl molecules^37,38^ while the TD-LC-DFTB scheme has been tested and used earlier for Chl molecules of bacterial LH complexes.^38^ The results clearly show that the numerically efficient TD-LC-DFTB formalism yields energies that are as accurate as the numerically much more expensive TD-DFT schemes. Therefore, they enable us to calculate excitation energies along a QM/MM MD trajectory sampling diverse conformations. All calculations have also been performed for the crystal structure geometry without lipids and solvents as a reference. The resulting energy ladder is presented in Figure S4. The relative excitation energies show a similar trend for both the QM/MM-minimized and crystal structure. Moreover, due to the existence of large thermal fluctuations in biological systems and in light-harvesting complexes,^39−42^ we proceed to determine the energy ladder within the FCP complex as an average over an ensemble of conformations. To this end, we have performed a 1 ns-long QM/MM MD simulation. Then, excitation energy calculations were carried out for the resulting conformations to determine the energy fluctuations along this QM/MM MD trajectory. The DFTB3 method with the 3OB-f parameter set was utilized for the ground state simulations, whereas the TD-LC-DFTB scheme with the OB2 parameter set was employed for the excitation energy calculations within the QM/MM framework. Details of this simulation are given in Materials and Methods in the Supporting Information. The associated excitation energy distributions (or densities of states, DOS) are shown in Figure S5. The sampling over different conformations along QM/MM MD trajectories on the DFTB level supports the finding that the Chl-c pigments have lower excitation energies than the Chl-a molecules within the FCP protein. These findings strongly suggest that the excitation energy within this FCP complex can be funneled toward the Chl-c2 pigment; i.e., an energy transfer from the Chl-c2 pigment toward the Chl-a pool is, on average, not always possible.

**Figure 2:**
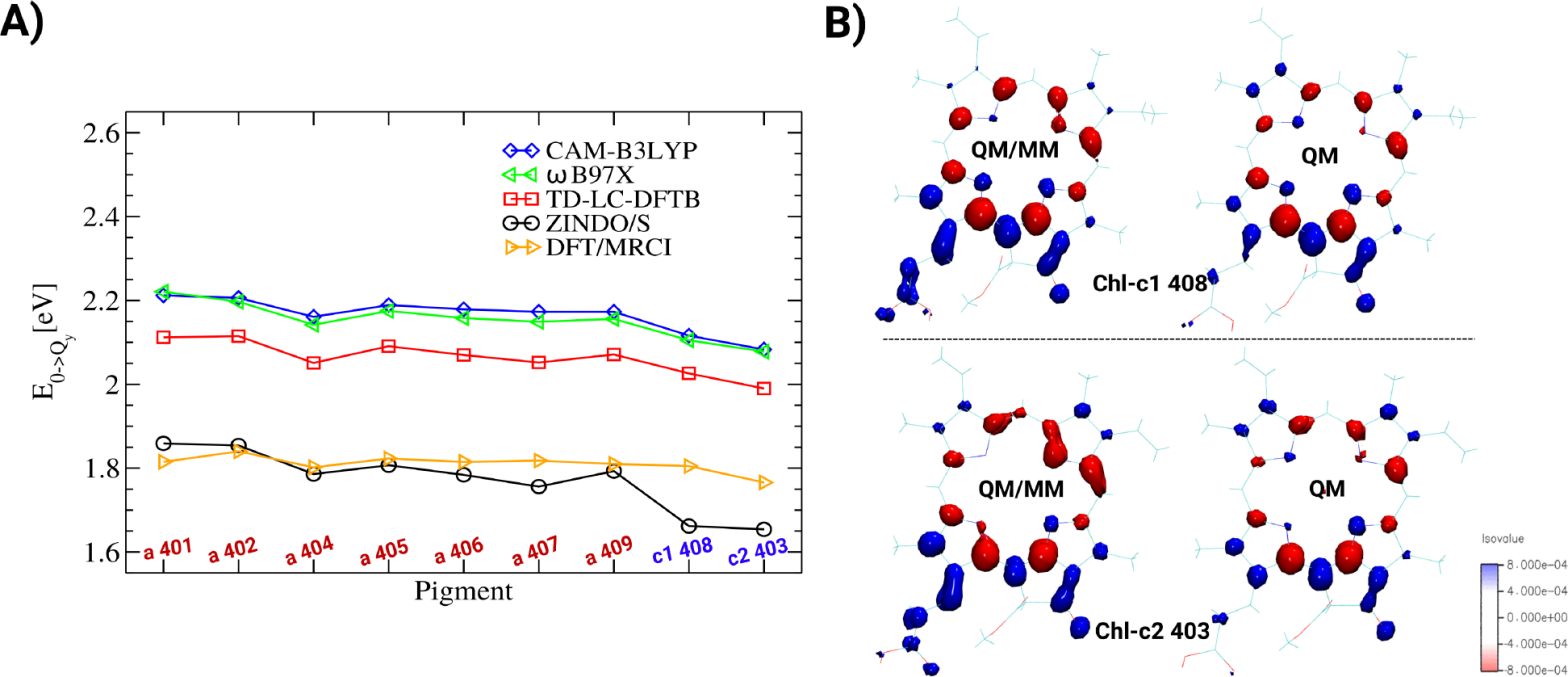
A) Q_*y*_ excitation energies of the FCP complex of the diatom *P. tricornutum* based on various levels of quantum chemistry. All calculations were based on the QM/MM-optimized structure. B) Density difference between the ground and the Q_*y*_ excited states for the Chl-c1 (upper panel) and Chl-c2 molecules (lower panel). The excited-state calculations were performed with and without coupling to the MM charges.

The results from different levels of theory shown in Figure 2A and listed in Table S1 agree with the fact that the Chl-c2 pigment has the lowest Q_*y*_ excitation energy. Moreover, the pigment Chl-c1 has the second-lowest energy, except for the DFT/MRCI calculation of the QM/MM optimized geometry. This finding is unexpected, since the Q_*y*_ excitation energies of Chl-c molecules in vacuum and in the organic solvents acetone and ether are blue-shifted with respect to those of Chl-a molecules.^16,28^ To decipher this energy ordering in FCP and to gain insight into the associated discrepancy in organic solvents and vacuum, we optimized the geometries of the Chl-a and Chl-c1/c2 pigments in the gas phase using the B3LYP level of theory together with the Def2-TZVP basis set. Subsequently, TD-LC-DFTB, TD-LC-DFT (CAM-B3LYP/Def2-TZVP), and DFT/MRCI calculations were performed. Furthermore, we have performed simulations of individual Chl-a and Chl-c1/c2 molecules in the solvents diethyl ether and acetone. One nanosecond-long QM/MM MD simulations were followed by TD-LC-DFTB calculations within the QM/MM scheme to obtain excited state energy distributions. The results of single-point calculations on the gas phase-optimized geometries as well as the averages along the QM/MM MD trajectories are presented in Table 1 together with experimental findings from the literature.^28^ The excitation energy distributions determined along the QM/MM MD trajectories with the solvents diethyl ether and acetone are shown in Figure S8. The data clearly show that Chl-c1 and Chl-c2 molecules have higher Q_*y*_ excitation energies compared to those of Chl-a molecules, both in theory and experiment. Since acetone is a more polar solvent than diethyl ether, one can expect a slightly larger directed electric field at the position of the pigment molecules due to specific solvent arrangements, especially in the first solvation shell. Therefore, in the solvent acetone the excitation energy distributions belonging to the Chl-c molecules are only slightly blue-shifted compared to those of the Chl-a molecules (Table 1 and Figure S8). Though qualitative differences exist for the individual approaches, the Q_*y*_ excitation energies in vacuum and organic solvents for the Chl-c molecules are always higher than those for the Chl-a pigments. Nevertheless, as shown in Table 1, a high-level quantum method such as DFT/MRCI can reproduce a quantitative blue shift of around 100 meV in the excitation energies of Chl-c with respect to Chl-a molecules, in line with experimental measurements.

**Table 1:** Q_*y*_ Excitation Energies (in eV) of the Chl-a and Chl-c1/c2 Molecules Based on a Gas-Phase Optimized Conformation as Well as the Averages Obtained Along QM/MM MD Trajectories with the Solvents Diethyl Ether and Acetone^a^

	Chl-a	Chl-c1	Chl-c2
TD-LC-DFTB (gas phase)	2.197	2.249 (+0.052)	2.224 (+0.027)
TD-LC-DFT (gas phase)	2.239	2.291 (+0.052)	2.262 (+0.023)
DFT/MRCI (gas phase)	1.910	2.023 (+0.113)	1.987 (+0.077)
QM/MM MD (ether)	2.065	2.082 (+0.017)	2.073 (+0.008)
QM/MM MD (acetone)	2.049	2.060 (+0.011)	2.050 (+0.001)
experiment (ether)	1.873	1.981 (+0.108)	1.977 (+0.104)

a The experimental results obtained in diethyl ether are shown for ref (28). The values in parentheses denote the differences with respect to the results obtained for Chl-a using the same approach.

To better understand these findings, one can also have a look at the structural differences between the Chl-a and Chl-c1/c2 molecules as depicted in Figure S1 with the Chl-c1/c2 molecules lacking the phytyl tail of Chl-a. Furthermore, the presence of an extra unsaturated pyrrole ring within the Mg-porphyrin chlorin ring and an acrylic group connected to the chlorin ring hamper the π–π conjugation in the Chl-c molecules. These structural differences are the main reason behind the blue shift of the Q_*y*_ excitation energy of the Chl-c1/c2 molecules in the gas phase or organic solvents. At the same time, the Chl-c1 and c2 pigments differ in the side chains of the Mg-porphyrin rings with a very small effect on the Q_*y*_ excitation energy, thus leading to similar energies for these two types of Chl-c molecules. The relative ordering of the Q_*y*_ excitation energies for Chl-a, c1, and c2 is identical in theory and the experiments as detailed in Table 1. The DFT/MRCI approach yields the most accurate values for the absolute and relative Q_*y*_ excitation energies compared to the experimental outcomes. Most interestingly, the order of the excitation energies for Chl-a, c1, and c2 is different between the gas phase (or organic solvents) and the FCP complex of *P. tricornutum* as shown in Figure 2A, where the Chl-c2 molecule has been found to be the most red-shifted pigment within this Chl network.

At this point, we sought to understand the effect of the electrostatic environment (protein, lipids, solvent, and ions) on the electronic structures of the chlorophyll molecules. To this end, we have calculated the density difference between the ground state and the Q_*y*_ excited state of the Chl-c molecules as shown in Figure 2B with and without QM/MM coupling. For other chromophores, we refer to Figure S11. The lower panel of Figure 2B shows a significant density delocalization on the Mg-porphyrin ring of the Chl-c2 molecule when the system is treated within the QM/MM framework compared with the calculations without QM/MM coupling. This density difference is consistent with the large shift in the Q_*y*_ excitation energy of Chl-c2 pigment 403. For Chl-c1 pigment 408, a similar effect can be observed in the upper panel of Figure 2B. For this pigment, the change in the difference density with and without QM/MM coupling is, however, not as large as in the case of the Chl-c2 molecule, especially near the NC atom of the Mg-porphyrin ring. Therefore, it is reasonable that the difference in the Q_*y*_ excitation energy is also not as large as that found in the energy ladder of the FCP complex. Please note that along the QM/MM MD trajectory only Chl-c2 has the lowest average excitation energy in the energy ladder as shown in Figure S5 due to the fact that Chl-c2 experiences a stronger electric field as shown below. For the Chl-a pigments, much smaller effects of the electrostatic environment on the difference density can be noticed in Figure S11. This fact also agrees with the former analysis in which the excitation energy shifts for the Chl-a pigments along the 1 ns QM/MM MD trajectory and for the QM/MM-optimized geometry are smaller when comparing the results with and without QM/MM coupling in Figure S7. Nevertheless, for LH complexes containing only Chl-a pigments, these small changes are important for forming the energy ladder in those systems. Moreover, the finding of large difference densities for the Chl-c1 and Chl-c2 pigments corroborates the unexpected energy shifts for these pigments within the FCP complex of the diatom *P. tricornutum*.

To better understand the effect of electric fields on the excitation energies of Chl-a and Chl-c molecules, we studied these chromophores in the gas phase under the influence of electric fields in different directions. To this end, we have first aligned the gas phase-optimized pigments in the *x* – *y* plane by moving the nitrogen atoms to a position very close to the *z* = 0 plane. Moreover, we have rotated the molecule in the *x* – *y* plane such that a maximum effect of the electric field in the *y* direction is observed. Subsequently, seven calculations were performed for each chromophore, i.e., no electric field as well as fields in the ±*x*, ±*y*, and ±*z* directions. The field strength in all cases was set to 0.01 in atomic units, i.e., *e*/*a*_0_^2^, corresponding to 5.14 V/Å. TD-DFT calculations with the CAM-B3LYP functional and the Def2-TZVP basis set were performed for all excited state calculations as implemented in the ORCA program.^43^ The results are listed in Table 2, and the associated density differences are depicted in Figure 3 for fields in the positive *x*, *y*, and *z* directions. For the fields with opposite directions, the excitation energies and the associated density differences are shown in Table 2 and Figure 3, respectively.

**Table 2:** Q_*y*_ Excitation Energies (in eV) of the Chl-a and Chl-c1/c2 Molecules Based on a Gas-Phase-Optimized Conformation and in the Presence of Homogeneous Electric Fields in Directions as Indicated and Described in the Text^a^

	Chl-a	Chl-c1	Chl-c2
no field	2.243	2.289	2.263
+*x* direction	2.192 (−0.051)	2.252 (−0.037)	2.248 (−0.015)
+*y* direction	2.268 (+0.025)	2.037 (**−0.252**)	2.035 (**−0.228**)
+*z* direction	2.243 (0.000)	2.287 (−0.002)	2.241 (−0.022)
–*x* direction	2.249 (+0.006)	2.295 (+0.006)	2.243 (−0.020)
–*y* direction	2.220 (−0.023)	2.285 (−0.004)	2.242 (−0.021)
–*z* direction	2.239 (−0.004)	2.287 (−0.002)	2.278 (+0.015)

a The values in parentheses show the difference with respect to the results without a field. The maximum change in the excitation energy of the Chl-c molecules is found for the field in the +*y* direction.

**Figure 3:**
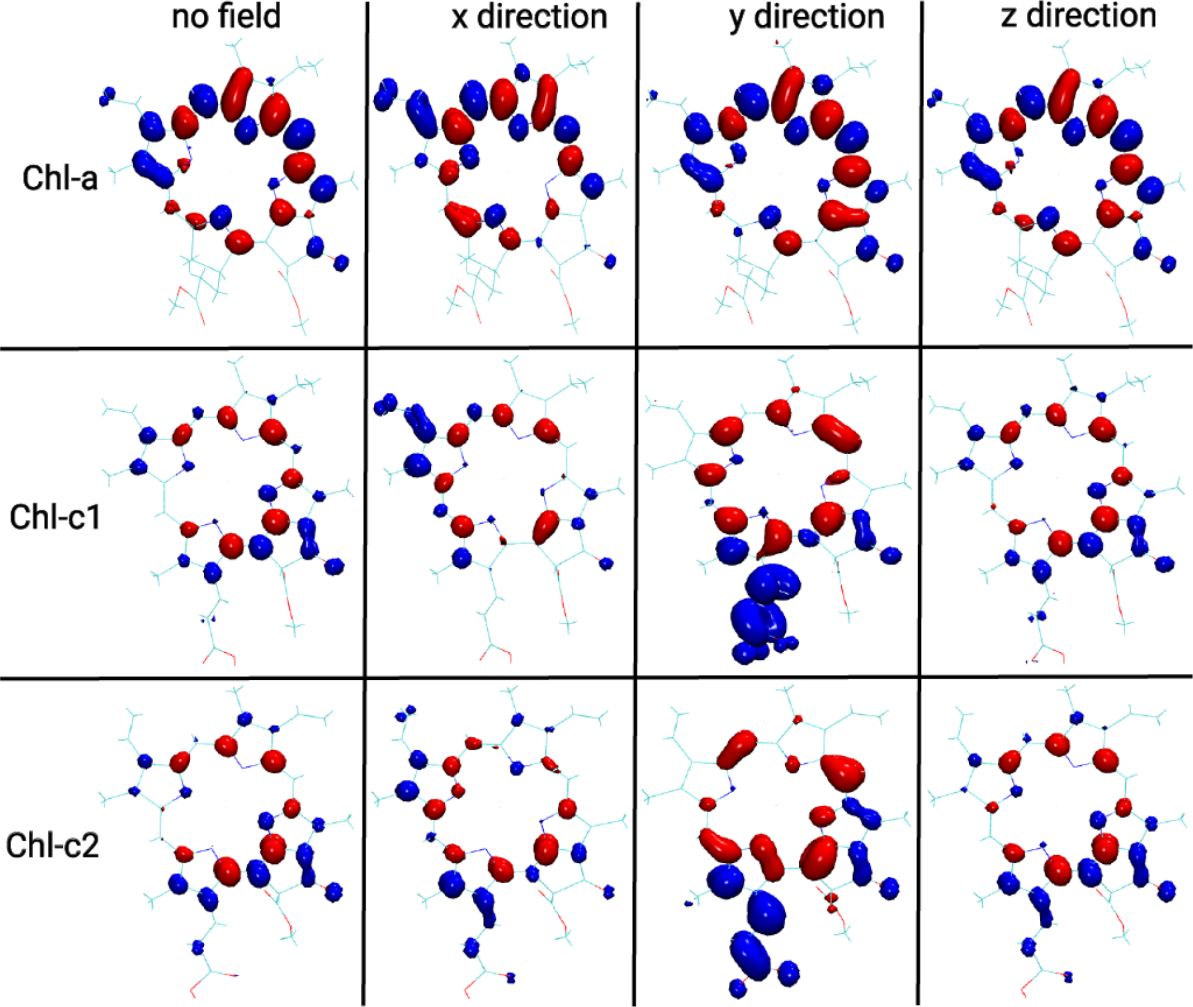
Density differences between the ground and excited Q_*y*_ states for the Chl-a and Chl-c1/c2 molecules without and with an external electric field with a strength of 0.01 au along the positive *x*, *y*, and *z* directions.

As one can see from Table 2, the excitation energies for the Chl-a molecule are affected by homogeneous fields in the *x* and *y* directions and slightly in the *z* direction. For an electric field in the +*x* and +*y* directions, there are noticeable shifts by −0.051 and 0.025 eV for the Q_*y*_ excitation energies, respectively. For the opposite field directions, the largest shift is found for the field in the negative *y* direction with an energy shift of −0.023 eV. Such energy variations within electric fields produced by the protein environment are actually responsible for the energy ladders in LH complexes such as LHCII,^44^ CP29,^45^ and CP43.^46^ The picture is, however, different for the Chl-c molecules. Although the pigments again mainly react to electric fields along the *y* axis (as assigned here), there is almost no effect in the negative *y* direction and a very large effect in the positive *y* direction reducing the energies by −0.252 and −0.228 eV for Chl-c1 and Chl-c2 molecules, respectively. Physiologically, this outcome is very important for the studied FCP complex since the protein surroundings of the Chl-c pigments reduce the excitation energies of these molecules below those of the Chl-a pigments for the studied case. Thus, the energy flow will take place on average from the Chl-a to the Chl-c chromophores, i.e., different from what is expected based on the excitation energies in the gas phase and organic solvents.^16,28^ These large shifts in the excitation energies are also reflected in the density differences between ground and excited states shown in Figure 3 for the positive field direction and in Figure S12 for the negative field direction.

Considering the chlorophyll pigment molecules within the FCP protein, the surrounding charged residues do create very specific electric fields at the positions of the chromophores. We have selectively calculated the electric fields at the positions of the magnesium and nitrogen atoms for the Chl-c1 and Chl-c2 pigments as test cases. To this end, we have aligned the pigment with the QM/MM-optimized geometry of the same pigment type such that the studied pigment lies basically in the *x* – *y* plane with the same orientation as the gas phase molecules. The respective environments of the pigments were moved accordingly. Subsequently, the electric fields produced by these environments have been measured using the TITAN code^47^ and are listed in Table 3. As can be seen, the fields at the different atomic positions can considerably vary in magnitude and direction within one molecule. Due to the above-described test calculations with homogeneous electric fields, we know that the Chl-c molecules are quite sensitive to fields in the positive *y* direction. Interestingly, at the positions of the NA and ND atoms and also at the position of the magnesium atoms, (strong) field components in this direction are present. As can be seen in Figure 3, these atoms are in those parts of the molecules that show the largest density differences. Moreover, the positive *y* components of the electric fields are about double in magnitude for the Chl-c2 molecules compared to those for the Chl-c1 molecules. These field differences explain that while the energy shifts for the two types of Chl-c molecules can be very similar in the case of the test fields above, the energy of the Chl-c2 molecule within the FCP protein is the lowest of all chlorophyll molecules.

**Table 3:** Example of Electric Fields (in Atomic Units) Acting at the Magnesium and Nitrogen Atoms of the Chl-c1 and Chl-c2 Pigments within the FCP Complex^a^

	*x*		*y*		*z*	
	Chl-c1	Chl-c2	Chl-c1	Chl-c2	Chl-c1	Chl-c2
MG	–0.0056	–0.0016	**0.0003**	**0.0031**	0.0354	–0.0102
NA	–0.0020	0.0032	**0.0115**	**0.0220**	0.0049	0.0011
NB	0.0055	0.0178	–0.0002	–0.0055	0.0056	–0.0044
NC	–0.0085	–0.0075	–0.0057	–0.0170	0.0054	–0.0034
ND	–0.0108	–0.0193	**0.0021**	**0.0103**	0.0033	0.0003

a The field components in the *y* direction, which have positive values, are given in boldface.

To model the absorption spectra of LH systems from an atomistic point of view, the key quantities are the site energies, excitonic couplings, spectral densities, and transition dipole moments. Redfield-like approaches have been employed to determine the linear absorption of LH complexes. Recently, Cao and co-workers have developed the full second-order cummulant expansion (FCE) to calculate the linear absorption, an approach that includes neither the Markovian nor the secular approximation.^48,49^ The FCE method has been found to overcome several shortcomings of Redfield-like methods.^46,50^ Here, we have employed a code provided by Cupellini and Lipparini to determine the absorption spectra based on the FCE method.^51^ No information on the static disorder can be extracted from relatively short time trajectories, and thus no static disorder is included in the calculations of the absorption spectra.

The spectral densities of individual pigments were extracted and included in the SI, alongside the experimental data obtained from the LHCII complex of the higher plant. Calculated spectral densities using the present approach have been compared to their experimental counterparts for several light-harvesting complexes.^41,44−46^ The determination of the absorption spectra based on these spectral densities gave mixed results.^45,46^ Thus, we pursue two options in the following text: we determine the absorption spectra with the calculated spectral density as well as with the experimental one, being aware that it has been determined for a slightly different complex. In both cases, the time-averaged Hamiltonian shown in Table S3 has been employed. In the first set, the experimental spectral density has been considered, and the comparison between simulated and experimental absorption spectra is presented in Figure 4A. The computed spectrum has been shifted by about −1049 cm^–1^ toward lower energies with respect to the experimental one in order to align the maximum peak. As one can see, the overall line shape of the calculated spectrum is in reasonable agreement with the experimental counterpart. Moreover, we repeated the calculations neglecting the high-frequency parts of the Chl-a and Chl-c spectral densities above 800 cm^–1^ stemming from the intramolecular vibrations to gain insight into the origin of the sideband. As can be seen in Figure 4A, the sideband disappears, making it obvious that it is due to the vibrational progression of the Chl-a molecules and is not due to absorption peaks of Chl-c pigments as assumed earlier in the literature.^16,17^ We note in passing that the latter spectrum was shifted by −1452 cm^–1^ with respect to the experimental one due to the change in the reorganization energy for this modified spectral density.^46,52^ In the second set of analyses, we have repeated the calculations based on the atomistic spectral density and give the results in Figure S16. In this case, the sideband is considerably higher than in the experimental spectrum, a phenomenon which we observed earlier in the case of the light-harvesting complex CP43 when using a spectral density determined using the same formalism as employed here.^46^

**Figure 4:**
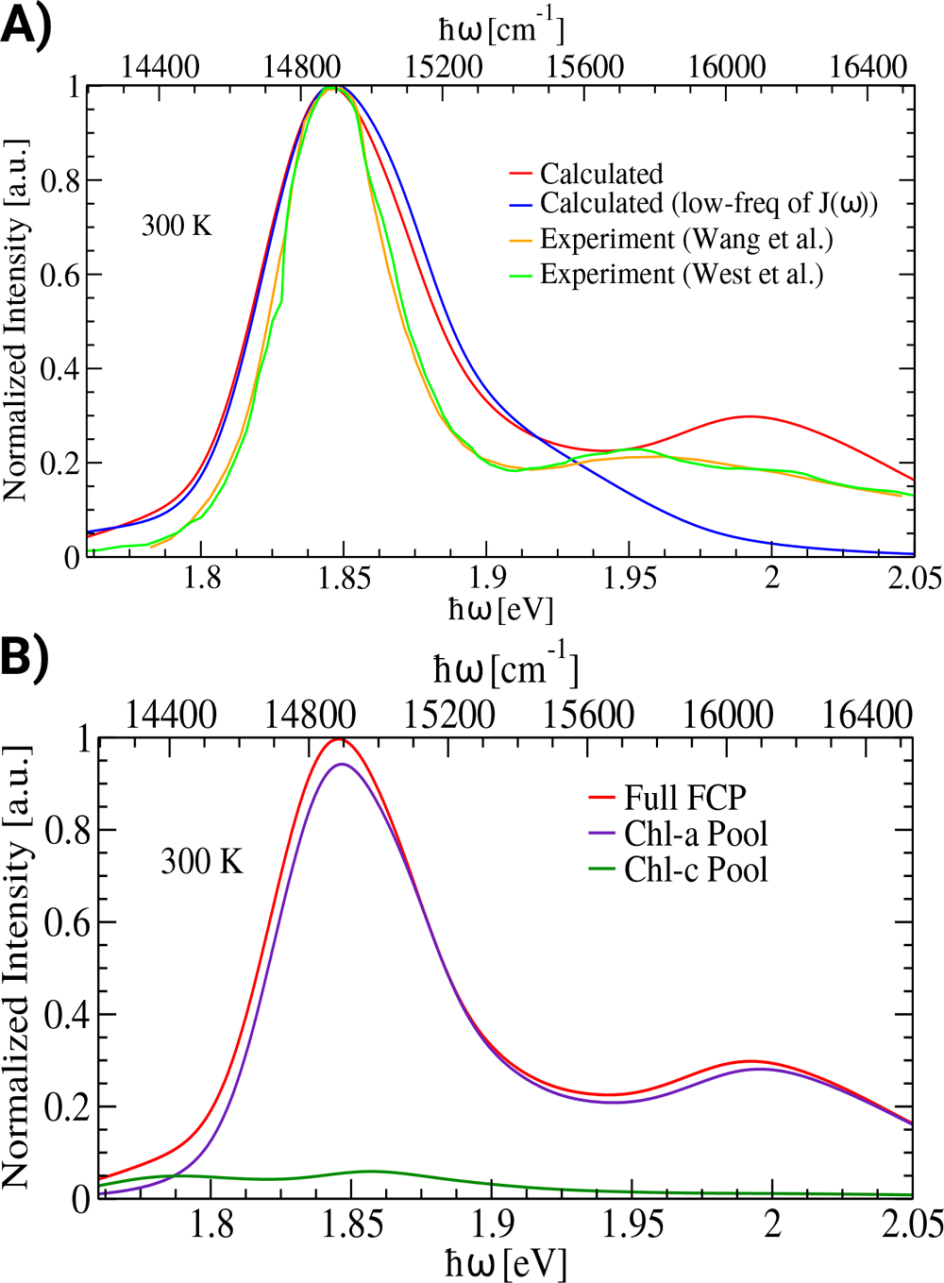
A) Calculated absorption spectrum of the FCP complex at 300 K (red) vs the experimental results (orange,^7^ green^33^). In addition, a test has been performed in which part of the spectral densities above 800 cm^–1^ has been neglected (blue). B) Contributions of the Chl-a (violet) and the Chl-c pigments (green) to the total absorption of the FCP complex (red).

From these calculations of absorption spectra, it becomes apparent that the high-frequency sideband of the absorption for frequencies larger than 15300 cm^–1^ (corresponding to roughly 1.9 eV) is a vibrational sideband stemming solely from the Chl-a molecules. To further understand the origin of the different parts of the absorption spectrum, we calculated the spectra taking into account only the Chl-a or only the Chl-c molecules. To this end, the Hamiltonian, spectral densities, and transition dipoles have been considered separately for the Chl-a and the Chl-c pool. Mainly, the Chl-a pigments contribute to the overall spectrum, including the main peak and the high-frequency sideband as shown in Figure 4B. This finding can be explained by the fact that Chl-c molecules have smaller transition dipole moments (and oscillatory strengths) compared to those of the Chl-a pigments and also by the low intensity of the high-frequency peaks in the spectral density as discussed earlier. Looking at these results, it seems clear that Chl-c molecules can contribute only minimally to the linear absorption spectrum of the FCP complex. However, we cannot exclude the possibility that under certain experimental conditions, e.g., excitation wavelength, purification method, etc., a population of Chl-c molecules exhibits absorption peaks consistent with the assignments in the experimental literature so far. At the same time, it is quite peculiar that in the experimental absorption spectra of FCP a small additional peak is visible in the range of 15576 to 15800 cm^–1^, i.e., roughly around 630 to 642 nm. This is surprising since this is the same frequency range found for the Q_*y*_ absorption of individual Chl-c molecules in organic solvents.^5,16,53^ Partially due to this coincidence, the small peak has been assigned to the Q_*y*_ excitation of Chl-c molecules also within the FCP complex.^16,17,30,31,54,55^ Based on the present results, however, we do believe that Chl-c absorption peaks have to be carefully evaluated and might be reassigned in future experimental and theoretical studies, given also the observation within this study.

In addition to the linear absorption, we have modeled the 2DES spectrometer, including a direct comparison to the experimental findings. Details are given in the Materials and Methods section in the Supporting Information. The 2DES for a selection of waiting times is shown in Figure 5A. The main features are in good agreement with those reported experimentally at room temperature.^5,16,17^ The absence of the weak excited state absorption peak above the main diagonal peak observed experimentally is an indication that the ratio between the couplings compared with the magnitude of disorder is somewhat underestimated. As in the experiment, a small time evolution of the main peak is seen, and the vibronic peaks are stretched out in both frequency directions from the main peak.

**Figure 5:**
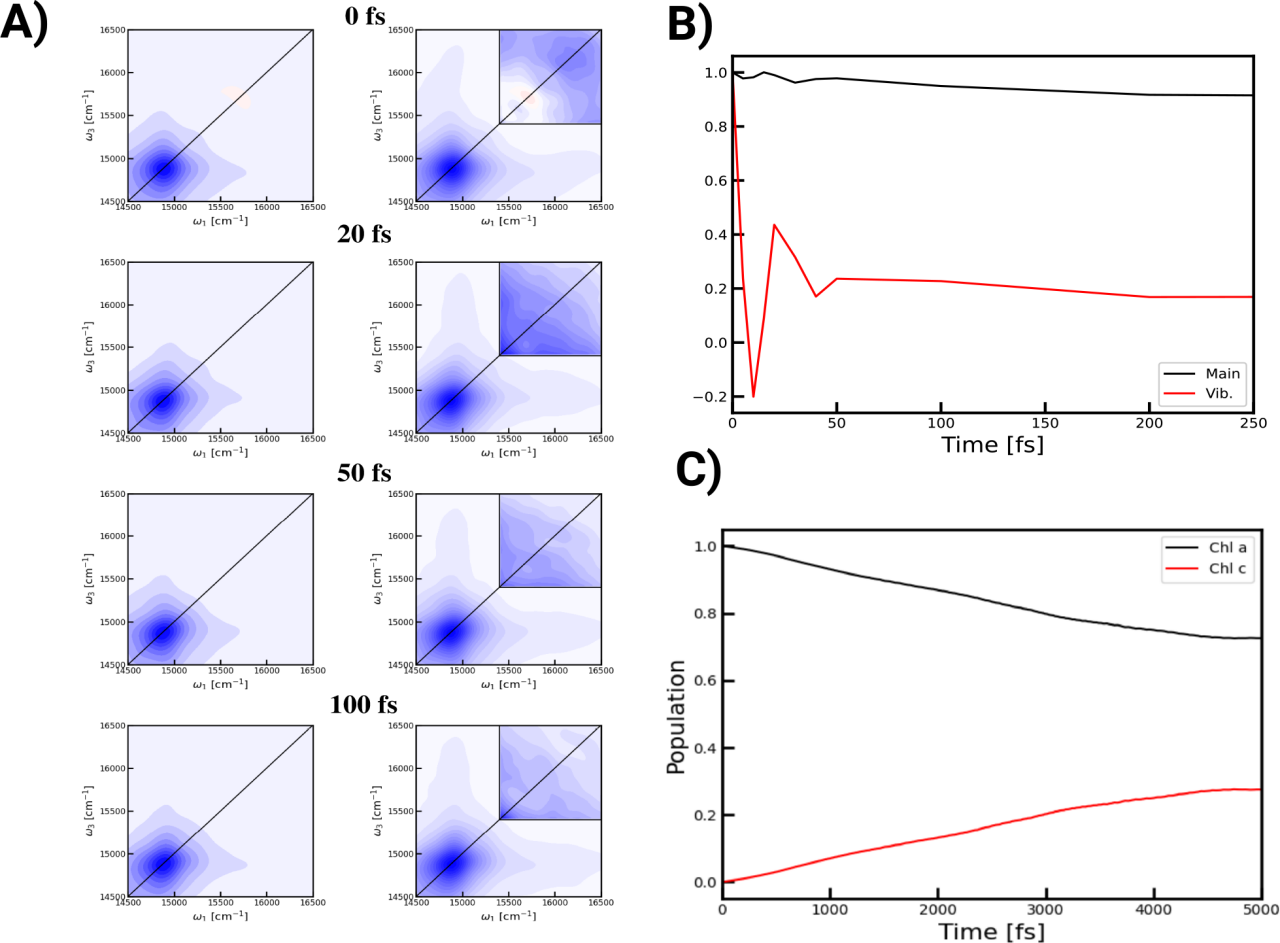
A) 2DES spectra with parallel polarization were obtained at 0, 20, 50, and 100 fs. The blue contours represent bleach-type signals, while red represents induced absorption. In the left column, the contour lines are plotted in different shades in steps of 10% of the maximum signal. In the right column, the same is true, but for the arcsin of the signal. The signal in the upper right square was further magnified by a factor of 20. B) Time traces normalized to the values at zero waiting time along the diagonal for the main peak region, i.e., 14800–15000 cm^–1^, and for the region of the vibronic peak, i.e., 16100–16400 cm^–1^. C) The population transfer has been calculated as the probability of starting at a Chl-a molecule and staying there vs the probability of ending up at one of the Chl-c pigments.

Normalized time traces of the diagonal main peak and the position of the diagonal vibronic peak are shown in Figure 5B. The time evolution of the diagonal main peak is very slow. The results are in good agreement with the experimental data^5^ and suggest that excitations of the main exciton transitions do not relax to excitons in a different region and that these excitons do not experience significant spectral diffusion on the subpicosecond time scale. In contrast, the dynamics of the vibronic peaks are very fast. Within 50 fs, the peak intensity decays to 20% of the initial value; i.e., a fast-damped oscillation is observed. In the experiment,^5^ the signal at this position exhibits a 50 fs decay. No oscillations are observed in the experiment; however, this is likely due to the finite time duration of the experimental laser pulse. Overall, the predicted time evolution of the two examined peak positions agrees well with the overall behavior observed experimentally. In the next step, we analyzed the energy transfer from the (according to the calculations shown above) energetically higher-lying Chl-a to the energetically lower-lying Chl-c chromophores. To this end, the energy transfer was determined using the NISE scheme while averaging over calculations in which the initial excitation was at any of the Chl-a chromophores. In Figure 5C, the resulting populations are shown. The transfer takes place on a picosecond time scale, which was to be expected due to the rather low excitonic coupling between the pigments. The largest coupling we calculate between a Chl-a and a Chl-c chromophore is on the order of 25 cm^–1^, corresponding to a transfer time of about 1.3 ps for the energy transfer between those molecules.

At this point, some comments are in order concerning the comparison to the experimental findings. In transient absorption measurements after the excitation of fucoxanthin in FCP with 530 nm pulses and probing at 634 nm, where Chl-c absorbs in organic liquids, no additional dynamics were found “that could be attributed to the transient population of an excited state of Chl-c”.^13^ This finding was subsequently confirmed in another experimental study concluding that “None of the results contained spectral signatures indicative for active involvement of Chl-c in the excitation energy transfer from Car to Chl a, i.e. the fx molecules transfer the energy directly to the Chl a molecules.”.^29^ These results are in line with our finding that the Chl-c molecules within the FCP complex are energetically not in between the fucoxanthin and the Chl-a energies, although the measurements were made for the slightly different diatom *Cyclotella meneghiniana*. Furthermore, in calculations trying to explain the experimental 2D spectra, it was noted that the “ultrafast energy transfer from the internal Chl-c state still remains an issue to be explained.”.^17^ Again, no structural data of an FCP complex were present at that point in time, although it was already clear that the excitonic couplings would be too small to allow for an exciton transfer from Chl-c to Chl-a within tens of fs. With the present assignment of the 2D peaks, no “issue” is left concerning the interpretation of these experiments, and the theoretical findings are consistent with previous measurements, albeit on a slightly different organism.

FCP proteins are the major LH complexes of marine photosynthetic diatoms that contribute large amounts to global biomass. Although the FCP complexes from diatoms belong to the same class of LH proteins as those of higher plants,^5^ their photophysical properties differ significantly because they contain different types and amounts of pigments. So far, FCP complexes have not been studied extensively from an atomistic point of view since the respective crystal structures from two different organisms (*P. tricornutum*^7^ and *C. gracilis*(8,9)) have been solved only recently. FCP complexes contain Chl-a/c and the carotenoids Fx and Ddx. On one hand, the Chl network needs to control the excitation energy transfer from the peripherical entities toward the reaction centers of the PSII/PSI complexes. On the other hand, carotenoids participate in the regulation of the photoprotective mechanism in the diatoms and can release excess energy as heat upon excess illumination on the turbulent ocean surface.

In vacuum or organic solvents, the Q_*y*_ excitation energies of the Chl-c molecules are blue-shifted compared to those of the Chl-a molecules. Hence, one could always expect a similar trend within the FCP complex, leading to an exclusive energy transfer from the Chl-c to the Chl-a molecules but not in the reverse direction. In fact, this trend has been proposed based on two-dimensional and pump–probe spectroscopy for the FCP complex of the diatom *C. meneghiniana*.^13,15−17^ However, the present study of an atomistic-structure-based analysis of the FCP complex from the diatom *P. tricornutum* reveals an alternative energetic ordering within FCP complexes. Based on a variety of quantum chemical approaches for single conformations as well as long QM/MM MD trajectories, we found that the Chl-c2 pigment 403 can have the lowest excitation energy in *P. tricornutum*. We found that electric fields in specific directions within the FCP matrix can substantially reduce the excitation energies of Chl-c molecules, about 5 times more than in Chl-a molecules. This electric field effect on Chl-c2 is the largest concerning the excitation energy as well as the density difference between the ground state and Q_*y*_ excited state. Thus, the peculiar properties of Chl-c molecules are the main reason behind the counterintuitive energy ladder in the studied FCP complex under the present simulation conditions.

Furthermore, we have determined the linear absorption spectrum based on atomistic simulations and found good agreement with the experimental measurement for most of the spectrum, but we did not find a specific peak that can be associated with the absorption of Chl-c molecules. Surprisingly, for our model setup, the contribution of the Chl-c molecules to the absorption is small compared to that of the Chl-a molecules, and their Q_*y*_ excitation energies cannot be directly determined from the absorption spectrum of the FCP complex. Potentially, previous assignments of the experimental Q_*y*_ excitation energies of the Chl-c pigments in FCP complexes might need to be reconsidered or explained in view of the new results herein.^13,15−17,29^ One has to keep in mind that some of the observed differences between theory and experiment might stem from varying experimental conditions or external stimuli as discussed below. As already stated, we cannot exclude the possibility that the experimental setup (excitation wavelength, purification methods, etc.) can introduce a population of Chl-c molecules with absorption peaks as assigned in the experimental literature. In order to enhance the comparison to experimental findings, we modeled 2DES spectra and compared them with the experimental measurements conducted on the FCP complex of the diatom *C. meneghiniana*.^13,16,17^ Our findings indicate that the signal, which exhibits a rapid decay within 50 fs as observed in the experimental measurements, can actually also be attributed to the vibrational dynamics within Chl-a rather than mainly to the exciton transfer from Chl-c to Chl-a as proposed in previous studies.^16,17^ Additionally, we have demonstrated that under certain conditions the transfer of excitons takes place from Chl-a to Chl-c pigments on a picosecond time scale and not from Chl-c to Chl-a in tens of fs. Importantly, these findings are consistent with the size of the excitonic couplings in the studied system. Further experimental data to be considered concern the fluorescence. This property has been reported in ref (7) for the organism *P. tricornutum*. A similar fluorescence line shape was also described for the organism *C. meneghiniana*.^56^ In this study, a terminal emitter energy of 676 nm was reported. This result is more difficult to reconcile with the outcomes of the present simulations. The question arises as to whether all experimental and theoretical investigations on the studied FCP complex have been performed under the same conditions. For example, a recent study has shown that the protonation state of the acrylate group in Chl-c molecules plays a major role in its characteristics.^27^ Apparently, a change in the respective protonation state can lead to substantial changes in the excitation energy and the transition dipole moment. Thus, potentially different protonation states of the Chl-c molecules could already explain some of the differences between theory and experiment mentioned above, especially concerning the energy ladder. Another possibility is that the protein matrix of the FCP complex is distorted under certain conditions, which might lead to different electric fields at the positions of the Chl-c pigments and less significant shifts in the excitation energies. These and other factors will have to be investigated with care in future theoretical and experimental studies.

The present study can be seen as a detailed structure-based analysis of the FCP complex of the diatom *P. tricornutum*, which asks for a more skeptical peak assignment in absorption spectra of the FCP complex and re-elaborates some previous interpretations of experimental results regarding the Chl-c pigments in FCP complexes, especially the proposed order in the energy ladder. The present outcomes further trigger the question of whether Chl-c pigments in FCP complexes from other organisms also have the lowest Q_*y*_ excitation energies or if the energetic ordering might be different due to varying protein environments. From an evolutionary point of view, it seems unlikely that the specific electric fields near the Chl-c pigments are not present in the FCP complexes from different organisms but might be switched on and off depending on the environmental conditions. Interestingly, when performing a computational experiment by removing the QM/MM coupling to the environment in the excited state calculations, the Chl-c2 pigment is found to have the highest excitation energy shift compared to the rest of the Chl both for the QM/MM-minimized geometry and for the average value determined along the QM/MM MD trajectory. This fact signifies the strong and peculiar effect of the FCP protein matrix on Chl-c properties of the diatom *P. tricornutum*. In this context, a recent experimental study on the FCP of *P. tricornutum* has been carried out to investigate the energy transfer from the Fx carotenoid to the Chl-a pool^33^ which possibly could be extended to investigate the energy transfer dynamics from Chl-c to Ch-a molecules. At the same time, a recently solved structure of the FCP complex from the diatom *C. meneghiniana*(12) needs to be analyzed in more detail in order to understand whether the FCP matrices from different organisms contain similar electrostatic effects on the Chl-c pigments. Moreover, the proposed crystal structure of the FCP from *C. meneghiniana* in ref (5) together with the estimated energy transfer rates from Chl-c to Chl-a might need to be revisited.

To this end, a structure of the complete PSII supercomplex of diatom *P. tricornutum* would be highly beneficial. In addition, it would be helpful to investigate if the energy landscape in the FCP complex changes in the transition from the light-harvesting to the photoprotective state since the electrostatic potential at the sites of the Chl-c molecules might change, leading to different excitation energies. Thus, future investigations of the available cryo-EM structure of the PSII supercomplex belonging to the diatom *C. gracilis*(9) could provide valuable insight.

In conclusion, we emphasize that the present investigation is a first attempt for a multiscale approach to be employed for the site energies of an FCP complex and can serve as a reference to model the excitonic properties of similar complexes in future studies.

Our study pinpoints the necessity of carefully considering the protein matrix effects when interpreting experimental results on Chl-c containing LH complexes. The fine-tuning of the Chl-c properties regarding the electric field can be exploited as a design principle for artificial systems. Thus, the conclusions of this study should apply to both natural and artificial LH systems.

## Data Availability

The data that support the findings of this study are available from the corresponding author upon reasonable request. The authors declare that the present research has mainly been produced with publicly available software, as also detailed in the Materials and Methods section in the Supporting Information

## Supporting Information Available

Materials and methods section, extra figures of excitation energy ladders and distributions, density differences, effects of external electric fields, excitonic couplings, and spectral densities (PDF)
