# Supplementary material for: Electric Field Susceptibility of Chlorophyll c Leads to Unexpected Excitation Dynamics in the Major Light-Harvesting Complex of Diatoms
Source: J Phys Chem Lett. 2024 Feb 27;15(9):2499–510. doi: 10.1021/acs.jpclett.3c03241 (PMC10926154; doi:10.1021/acs.jpclett.3c03241)
Supplement: Supplementary file 1 — jz3c03241_si_001.pdf [file jz3c03241_si_001.pdf]

SUPPLEMENTAL INFORMATION
Electric Field Susceptibility of Chlorophyll c Leads to an Unexpected Excitation Dynamics in the Major Light-Harvesting Complex of Diatoms

Sayan Maity,† Vangelis Daskalakis,‡ Thomas L. C. Jansen,¶ and Ulrich Kleinekathöfer*,†

†*School of Science, Constructor University, Campus Ring 1, 28759 Bremen, Germany*

‡*Department of Chemical Engineering, School of Engineering, University of Patras, 26504 Patras, Greece*

¶*Zernike Institute for Advanced Materials, University of Groningen, Nijenborgh 4, 9747 AG Groningen, Netherlands*

E-mail: ukleinekathoefer@constructor.university

S1 Materials and Methods

The initial geometry employed here is an equilibrated structure extracted from our previous study[1]. First, a geometry optimization has been performed within a QM/MM framework based on the 3rd-order SCC-DFTB (DFTB3) scheme[2] with the 3OB-f parameter set[3] for the QM region and the AMBER03 classical force field for the MM region including the protein, lipids, solvent, and ions[4]. We have employed the interface between DFTB+ and

GROMACS[5] to perform the QM/MM minimization using the steepest-descent algorithm. Moreover, we would like to point out that for a complex electronic system like a Chl molecule, it is very hard to reach the global minimum of the ground state geometry within a QM/MM fashion. Hence, it is not unlikely that the QM/MM-minimized structures presented here can potentially still deviate from the global minimum. Subsequently, the optimized geometries have been utilized for the excitation energy calculations also in a QM/MM setting based on various QM levels such as semi-empirical, LC-DFT functionals and multireference methods. For the Chl-a molecules, the QM region was reduced by truncating the phytyl tail at the so-called C1-C2 bond and caped with a hydrogen atom. The rest of the phytyl tail has been described as part of the MM environment together with the protein, solvent, ions, and the other pigments. This kind of QM/MM setup was employed successfully earlier for BChl-a and Chl-a/b molecules of bacterial and plant LH systems in order to reduce the computational cost[6–8]. Furthermore, as the Chl-c pigments do not contain phytyl tails, for these molecules the whole chromophore was considered as the QM region while the rest of the system was described by MM point charges. For the semi-empirical methods, we have considered the ZINDO/S-CIS and the TD-LC-DFTB level of theories. In ZINDO/S-CIS calculations, the 10 HOMOs and the 10 LUMOs have been considered for the active space. Subsequently, excited state calculations have been carried out using the time-dependent extension of the long-range corrected DFTB (TD-LC-DFTB) employing the OB2 parameter set[9]. As alternatives for the excited state calculations, we have furthermore employed the semi-empirical ZINDO/S-CIS method (Zerner's intermediate neglect of differential orbital method with spectroscopic parameters together with configuration interaction using single excitation) and higher level QM methods like the time-dependent long-range corrected DFT (TD-DFT using the CAM-B3LYP[10] and the ωB97X functionals[11]) and the DFT/MRCI approach[12]. Using individual structures has the advantage that one can test various high-level QM methods. The TD-LC-DFT calculations were performed based on the CAM-B3LYP and the ωB97X range-separated functionals together with the Def2-TZVP basis set.

Moreover, we have utilized the Tamm–Dancoff approximation (TDA)[13] and the Resolution of Identity (RI) approximation to accelerate the TD-LC-DFT calculations by considering RIJCOSX[14] for the Coulomb integral and Hartree-Fock exchange as well as def2/J as an auxiliary basis set as implemented in the ORCA program. The same RI approximation was also employed together with the B3LYP/Def2-TZVP level of theory in order to optimize the Chl-a and Chl-c molecules in gas phase. The ZINDO/S and TD-LC-DFT calculations were performed using the ORCA package[15] whereas the TD-LC-DFTB calculations have been performed using the DFTB+ package[16]. In total, 10 excited states have been calculated from which the Q_y excitation energies had to be extracted. For the multireference method, we have employed the DFT/MRCI formalism employing an interface to the ORCA package[12]. We followed a protocol like the one described in our earlier work in order to extract the Q_y excitation energies of the Chl molecules[6,17]. The Hamiltonian R2018 and the "standard" parameter set were employed with a 1.0 E_h threshold for the initial and the production runs with the requirement for 12 roots during the DFT/MRCI calculations. Furthermore, eight HOMO and six LUMO states were considered as active space, including single and double excitations. In addition, all calculations were repeated in a QM/MM setup using the crystal structure coordinates for each pigment.

In order to obtain a reasonable sampling of the FCP conformations, a 1.1 ns DFTB/AMBER MD simulation was carried out. The last 1 ns trajectory was used to extract a total of 10,000 frames for the excitation energy calculations. The QM/MM MD simulations were performed within the interfaces of the DFTB+ and GROMACS packages. Subsequently, the TD-LC-DFTB single point calculations were performed in a QM/MM fashion along those QM/MM MD trajectories within the DFTB+ package. Making use of the periodic boundary conditions in the QM/MM MD calculations, we have shifted for each frame individually the QM region into the center of the simulation box for the single-point TD-LC-DFTB calculations. This treatment removes artifacts in the non-periodic excited state calculations during the trajectory analysis[18]. As a result of the excited state calculations along the trajectory, one

obtains the Q_y excitation energy fluctuations from which the excitation energy distributions also known as DOS can be determined.

In case of the spectral density calculations, an 80 ps-long DFTB/MM MD simulation was performed in which the last 60 ps-long part was stored with a 1 fs stride. This produce results in 60,000 frames which were then utilized for TD-LC-DFTB calculations also in a QM/MM framework. Subsequently, the respective autocorrelation functions were determined from the excitation energy fluctuations. Finally, a half-sided Fourier transformation was performed to extract the spectral densities for the individual pigment molecule (see section S11).

The 2DES were calculated with the NISE method[19,20] following the general procedure discussed in Ref.[21]. For these calculations the 60 ps QM/MM MD trajectory was used. The average energies were adjusted to match the average energies found in the 1 ns QM/MM MD trajectory. The largest shift was a 146 cm^{-1} blue shift for Chl-a 405. Furthermore, static dynamics was added using a slow (10 ps correlation time) overdamped Browninan oscillator[22] for Chl-a 404, a 405, a 406, and Chl-c2 403 to increase the variance of the short trajectory to match that of the long trajectory. The largest component was 253.5 cm^{-1} for Chl-a 405 and 120.6 cm^{-1} was used for Chl-c2 403. Compared to the overall standard deviation of the those sites (900 and 500 cm^{-1}) this correction is relatively small and is not expected to affect the presented conclusions. The average couplings described in Table S3 were used along with the transition dipole moments obtained from the 60 ps long trajectory. For all spectral calculations using NISE, the 1 fs time step from the trajectory was used and spectra were sampled every 5 fs along the trajectory resulting in slightly less than 12000 realizations for each calculation. The maximal coherence time used in the calculations was 128 fs, ensuring sufficient spectral resolution. A 70 fs exponential apodization function[23] was used for noise suppression. For the 2DES spectra waiting times from zero to 20 fs were calculated at 5 fs intervals, then at 10 fs intervals up to 50 fs and further waiting times of 100, 200, and 5000 fs were obtained. The propagation was performed with the sparse coupling algorithm presented in Ref. 24 and for the 2DES spectra 24 CPUs were used (6 MPI tasks

each using 4 CPUs)[20]. As for the absorption spectra the 2DES spectra were subjected to a systematic frequency shift to match the experimentally observed peak position.

S2 Structural Differences between Chl-a/c Molecules

The chlorin ring of the Chl-a molecules contains three unsaturated and a saturated pyrrole ring, whereas in the case of Chl-c molecules the chlorin ring includes four unsaturated pyrrole rings. Moreover, the so-called "phytyl tail", which is commonly found in chlorophyll molecules of plants and bacteria, is not present in Chl-c molecules. In addition, an acrylic group is connected to the chlorin ring of the Chl-c molecules. These structural differences between Chl-a and Chl-c molecules are depicted in Fig. S1.

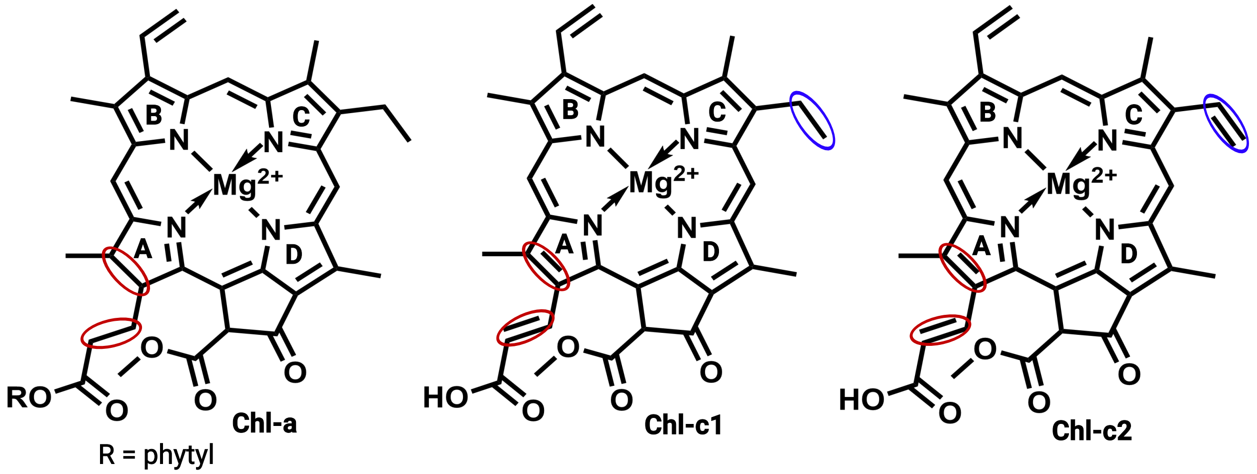

Figure S1: Structural differences between Chl-a and Chl-c1/c2 molecules. The red ellipses highlight the differences between Chl-c and Chl-a molecules, whereas the blue ellipses indicate the structural difference between the Chl-c1 and Chl-c2 variants.

Because of these structural differences, the intensity of the Q_y absorption is weaker for Chl-c than for Chl-a molecules[25,26]. In our calculations, the Q_y states have been identified based on their transition dipole moment orientation and direction. In case of Chl-a molecules, the NB-ND direction was considered as the direction of the transition dipole moment (see Fig. S1), whereas for Chl-c molecules the NA-ND direction was considered in accordance with the DFT/MRCI results as shown in the upper panels of Fig. S2 in the following section. The

selected Q_y state had the highest transition dipole moment strength as well as oscillatory strength along the time-dependent trajectory in line with the expected properties of the Q_y state. The Q_y state is usually the first excited state (S_1) for the Chl-a pigments. For the Chl-c molecules, however, the Q_y state jumps between the first (S_1) and second (S_2) excited states. An example for the behavior of the Q_y state for Chl-a and Chl-c molecules is shown in the lower panels of Fig. S2 in the subsequent section.

S3 Calculation of the Q_y Excited States

Subsequent to the excited state calculations, the Q_y excitation energies are extracted on the basis of the angle between the transition dipole moment of the respective state and the direction of two specific nitrogen atoms. To this end, the DFT/MRCI calculations which were carried for the gas phase-optimized structures of the Chl-a and Chl-c molecules were taken into account. After these calculations, the transition densities were extracted and fitted via the Multiwfn program[27] to determine the transition charges for each atom. We have considered the lowest excitation energy (S_1) among the first 10 excited states based on the experimental Q_y energy and a large transition dipole moment as well as oscillatory strength in the DFT/MRCI calculations. Subsequently, based on those atomic charges, the transition dipole moment has been calculated and visualized via the software VMD[28]. The upper panel of Fig. S2 delineates the length and direction of transition dipole moments for the different Chl molecules. Moreover, in this figure one can observe that the transition dipole moments are approximately directed along NB-ND axis for the Chl-a molecules whereas for the Chl-c molecules they are approximately aligned along the NA-ND axis. The atom names are based on the coordinates from the crystal structure. Once the Q_y states had been identified based on the DFT/MRCI calculations, the same approach has been applied for the other levels of quantum chemical calculations as considered in the present study. By doing so, we found that the Q_y states of Chl-a molecules are basically identical to the S_1 states

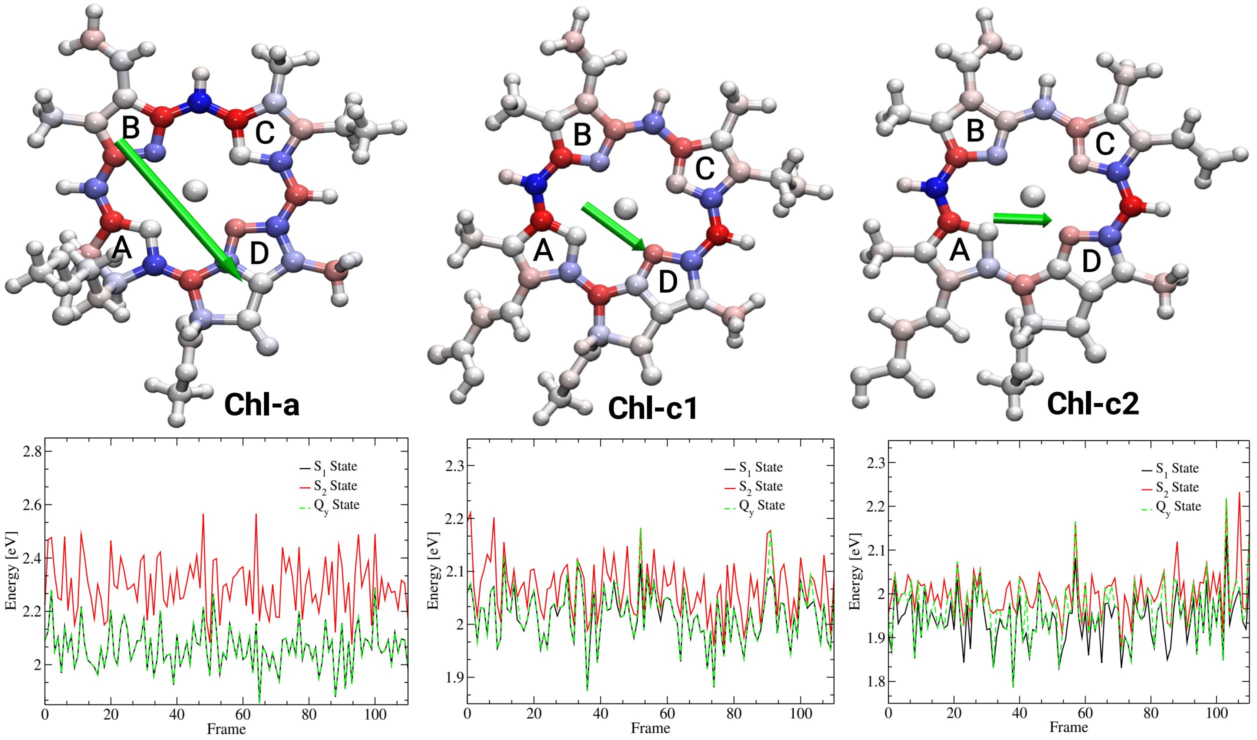

Figure S2: The upper panels depict the transition dipole moments of the Chl-a and Chl-c molecules in green based on the DFT/MRCI calculations on top the geometries optimized in gas phase. The lower panels show examples of the extraction of the Q_y excitation energies for Chl-a and Chl-c molecules based on the TD-LC-DFTB/MM approach along QM/MM MD trajectories on the basis of transition dipole moment orientation.

along the trajectory, whereas for Chl-c molecules the Q_y state jumps between the S_1 and S_2 states. Trajectory pieces of extracted Q_y energies along QM/MM MD trajectories based on TD-LC-DFTB/MM calculations are shown in Fig. S2.

S3.1 Electronic Nature of the S1/S2 Transition of Chl-c Molecules

Since the Q_y state of the Chl-c molecules jumps between the S_1 and S_2 states, we have further analyzed the electronic nature of these two states. The QM/MM optimized geometries were utilized and the density difference between the ground and the excited state have been extracted based on the TD-DFT CAM-B3LYP/Def2-TZVP level of theory as described in the main text. We have calculated the difference density with and without environmental point charges for the S_1 and S_2 excited states, as shown in Fig. S3. As one can see, both states

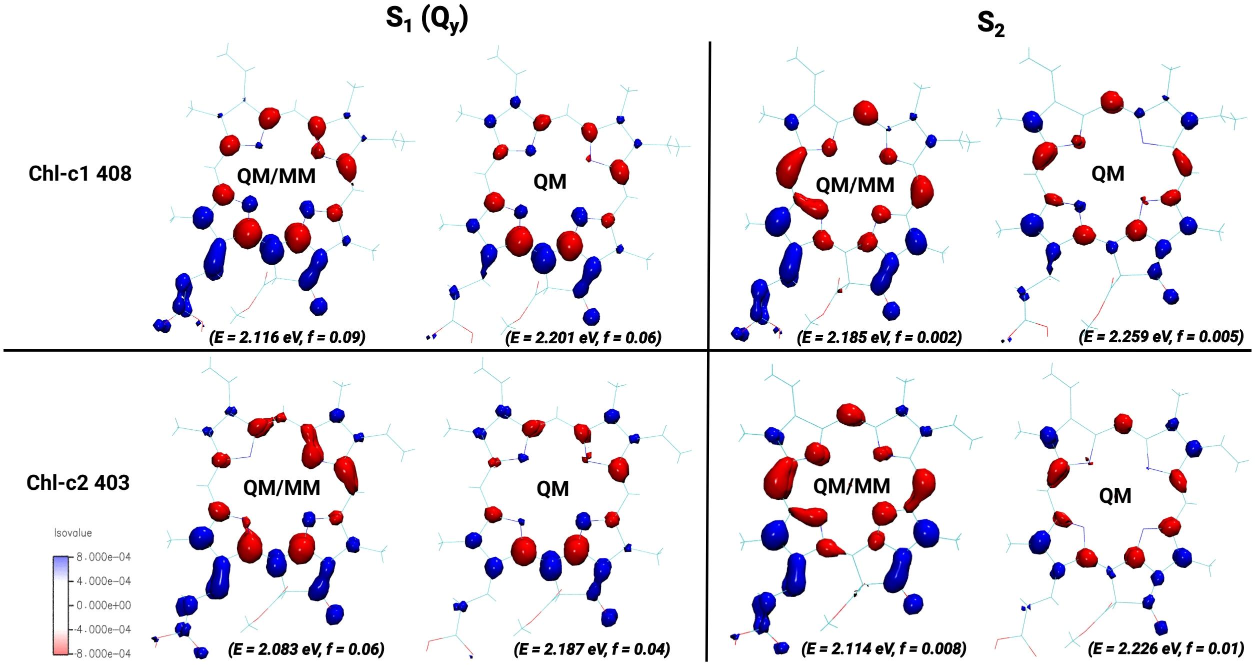

Figure S3: Density differences between the ground and the S_1 and S_2 excited states for the Chl-c1/c2 molecules. The calculations were performed with and without environmental charges. Moreover, the transition energies and its associated oscillatory strength are given in parentheses.

show a similar kind of electron density and a comparable effect of MM point charges. Hence, it is relatively easy that they trade their energetic positions along a trajectory as explained earlier. Once it has been identified if the S_1 and S_2 state corresponds to the Q_y state (see main manuscript), we determined the dot product between the dipole vectors of these two states. In this way, we found that the S_2 state of the QM/MM optimized structure has Q_x character for both Chl-c1 and Chl-c2 molecules. From the electron density delocalization shown in Fig. S3 one can also see that these two states do not have a charge transfer state. Moreover, the oscillatory strength of the S_2 is very low, as given in Fig. S3. Hence, it is likely that this state will not contribute significantly in the linear absorption of the FCP complex similar to the S_1 (Q_y) state as shown in the main text.

S4 Excitation Energy Ladder based on Crystal Structure

Fig. 2A of the main manuscript shows that the Q_y excitation energies based on the TD-LC-DFT method with either the CAM- B3LYP or the ωB97X functional and the Def2-TZVP basis set are quite similar. These energies are significantly higher than those for the DFT/MRCI or ZINDO/S approaches, which is a commonly known problem of (range-separated) DFT functionals as also shown earlier for Chl molecules of the LH complexes of bacteria and plants[6,8,18,29]. The DFT/MRCI method is based on a multireference scheme where a DFT calculation is performed at the beginning in order to construct the initial Kohn–Sham orbitals[30–33].

The Hamiltonian of the semi-empirical ZINDO/S has been parameterized among others for the equilibrium geometries of Chl-type molecules[34,35] and thus, as to be expected, its excitation energies lie within the range of the quite accurate DFT/MRCI calculations. In absolute terms, the energies of the parameterized TD-LC-DFTB method energetically lie below the LC-DFT results and above those from the DFT/MRCI and ZINDO/S schemes. At the same time, the TD-LC-DFTB findings maintain a very similar trend as the LC-DFT and the DFT/MRCI schemes at a significantly reduced computational cost.

In addition to the calculations for the QM/MM-optimized structure and along a QM/MM trajectory, we have computed the Q_y excitation energies of each Chl molecules based on the crystal structure conformations excluding lipids and solvents molecules. Sometimes these conformations from different LH complexes are used to benchmark excitation energies based on high-level methods and also assist to understand if some artifacts in the excitation energies can be caused by the lipids and solvent molecules around the pigments during the MD simulation. At the same time, we do believe that the QM/MM-optimized structure are more trustworthy to benchmark high-level quantum methods as explained in the main text. Fig. S4 depicts the excitation energy ladder of the FCP complex based on crystal structure as studied in the present work. The relative excitation energies maintain a similar trend as we have found for the QM/MM-optimized geometries. However, the excitation energies based

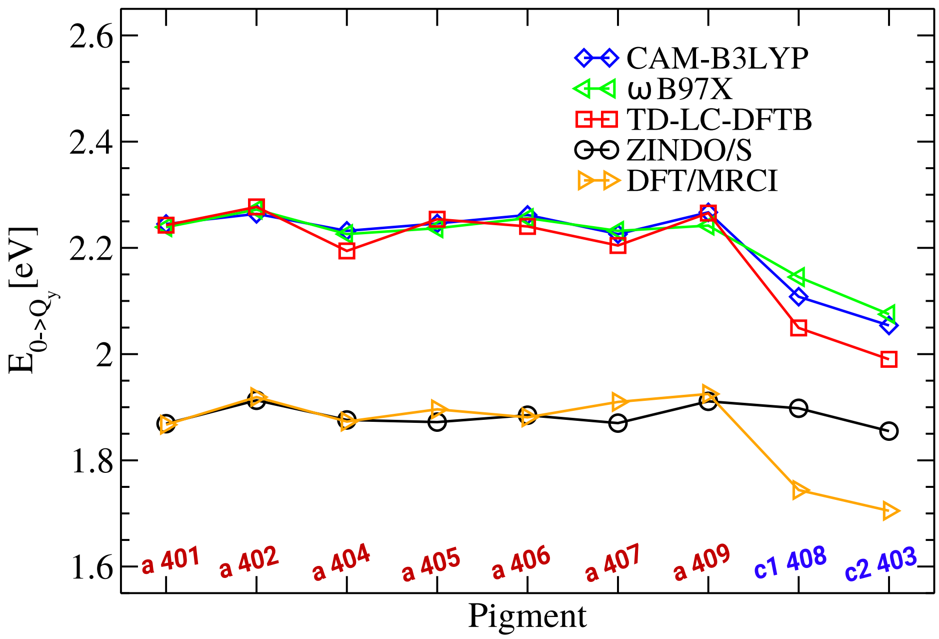

Figure S4: Same as shown in Fig. 2A of the main text, but based on the crystal structure.

on the DFT/MRCI method for the Chl-c molecules are significantly lower than those for the QM/MM-optimized geometries as shown in Fig. 2A in the main text. This finding is probably due to the fact that the inclusion of lipids and solvents in the QM/MM-optimized structures leads to different electrostatic environments of the Chl-c molecules compared to the crystal structure considered here. Moreover, the Chl-c2 pigments also have the lowest excitation energy in the energy ladder for all quantum chemical methods. Furthermore, the Chl-c1 pigment has the second-lowest excitation energy for all methods except for the ZINDO/S calculations. In case of the QM/MM-optimized structures, Chl-c1 has been the second-lowest excitation energy except for the DFT/MRCI calculations as tabulated in Table S1. Moreover, the Chl-c1 molecule did not show the second-lowest average excitation energy along the QM/MM MD trajectory. This is probably due to the fact that the y component of the electric field is not that large at the position of the Chl-c1 compared to Chl-c2 molecule, as listed in Table 3 of the main text.

S5 Excitation Energy Distributions along a QM/MM MD Trajectory

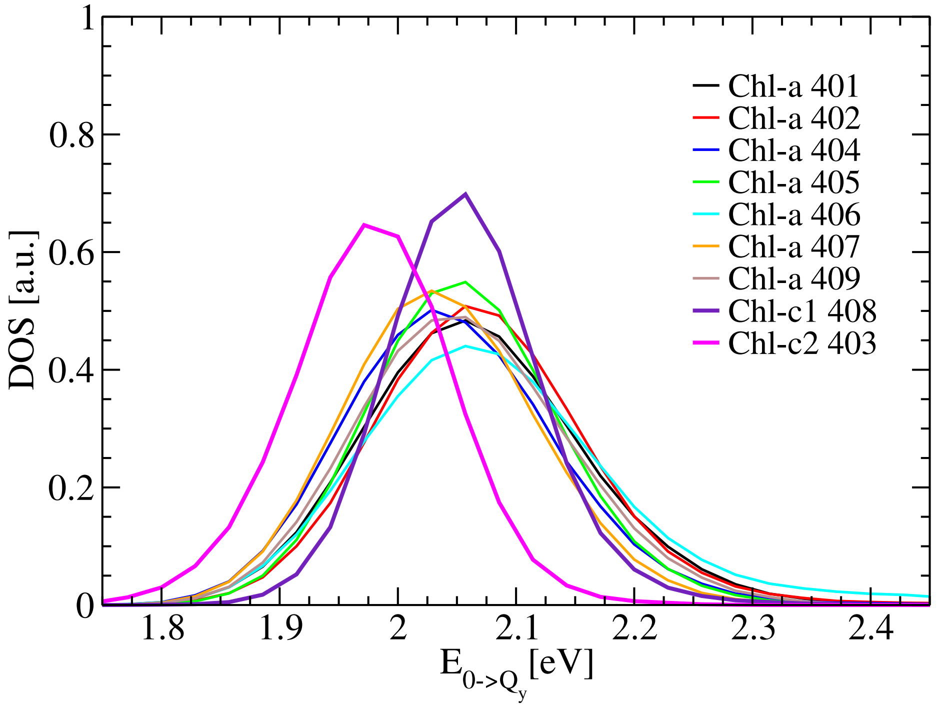

Figure S5: Q_y excitation energy distributions of the FCP complex from *P. tricornutum* based on a 1 ns-long QM/MM MD trajectory.

All excitation energy distributions (see Fig. S5) have a Gaussian shape and are shifted with respect to each other. From this figure one can again observe that the excitation energy distributions of the Chl-c2 pigment has clearly the lowest average excitation energy compared to all other chromophores. Thus, the average excitation energy of the Chl-c2 pigment as determined from the QM/MM MD trajectory has the same behavior as found for the QM/MM-optimized and the crystal structure geometries for various high-level QM methods as described earlier. The different levels of quantum chemistry approaches were also employed to rule out systematic errors in the excitation energies for the different pigment types and specific approaches. It is quite unlikely that the present results are due to an artifact present in all approaches at the same time. In recent experimental studies, pump-probe and two-dimensional spectroscopy has revealed an ultra-fast energy from the Chl-c2

pigments towards the Chl-a pool[25,36,37]. These experiments were made, however, for the diatom *C. meneghiniana* with a different (structurally so far unresolved) FCP complex than the one probed herein. Thus, no direct comparison can be made, but a re-analysis of these experimental findings might be in order.

To distinguish the effects of the environment and those of the internal conformations of the pigment molecules, used the conformations from the QM/MM MD trajectory and performed TD-LC-DFTB calculations for the excited states neglecting the effect of the environment, i.e., without the MM point charges of the environment in the excited state calculations. The resulting energy distributions without QM/MM coupling in the excited state calculations along the QM/MM MD trajectory are shown in Fig. S6. Clear differences between the latter and the distributions in Fig. S5 are evident. The same is true when comparing the average excitation energies with and without QM/MM coupling in the excited state calculations as can be observed in Fig. S7. Although the blue-shift in the Q_y excitation energies for the Chl-c molecules based on the TD-LC-DFTB method along the QM/MM MD trajectories without protein environment is not as large as found in organic solvent in experiment, one has to keep in mind that sampling over multiple conformations can only be achieved for a low-level quantum method such as TD-LC-DFTB. On one hand, such low-level theory can provide a qualitative trend along a trajectory whereas on the other hand, high-level quantum method such as DFT/MRCI can be used to obtain a quantitative benchmark based on single conformations as shown in Table 1 and Fig. 2A in main text as well in Fig. S4 for the gas-phase as well as inside a protein environment for the QM/MM-optimized and the crystal structures. Moreover, based on the QM/MM-optimized structures and shown in Fig. S6 the DFT/MRCI method without protein environment is yielding a similar blue-shift as the experimental values in organic solvents (see Table 1 in main text). The largest differences for the average energies can be observed in the case of the Chl-c2 molecules, while the second-largest energy shifts are observed for the Chl-c1 pigments. These findings again strongly suggest that the environment is playing a key role in the control of the Q_y

excitation energies of the Chl-c pigments and significantly affects the energy ladder within the present FCP complex. As an additional point, we have investigated the role of the local protein environment around the Chl-c molecules to observe whether any major structural change can potentially induce artifacts in the calculation of the excitation energies. However, as explained in Section S7, the small RMSD values of the C-α atoms belonging to nearby amino acid residues as well as of the Chl-c1/c2 pigments rule out this possibility (see Fig. S9 in the following section.).

S5.1 Excitation Energies Distributions without MM Environment

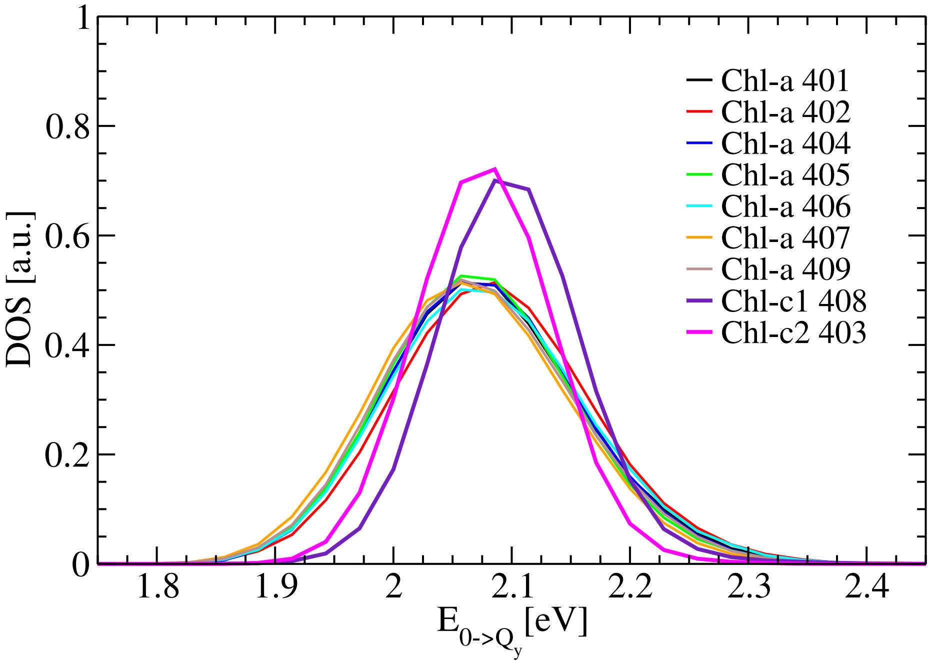

Figure S6: Excitation energy distributions of the FCP complex along the 1 ns QM/MM MD trajectory based on the TD-LC-DFTB approach, but without taking the QM/MM coupling into account in the excitation energy calculations.

As explained in the main text, Chl-c2 is the most red-shifted pigment in the energy ladder of the FCP complex. To better understand the reasoning for this shift, we repeated the TD-LC-DFTB calculations over the 1 ns QM/MM MD trajectory while neglecting the QM/MM coupling in the excited state calculations. The corresponding energy distributions are shown

in Fig. S6. From this figure, it can clearly be seen that the distribution of the Chl-c2 as well as the Chl-c1 molecules are clearly blue-shifted. Furthermore, in Fig. S7 we have presented the TD-LC-DFTB average excitation energies without the QM/MM coupling together with the average excitation energy calculated taking the MM point charges into account along the 1 ns QM/MM trajectory. In addition, the TD-LC-DFTB results for the QM/MM-minimized conformations are included. Furthermore, we have calculated the excitation energies without QM/MM coupling based on the CAM-B3LYP and DFT/MRCI approaches for the QM/MM-minimized structures and presented them in Fig. S7 together with the excitation energies calculated including the QM/MM coupling at the same level of theory. As one can see in

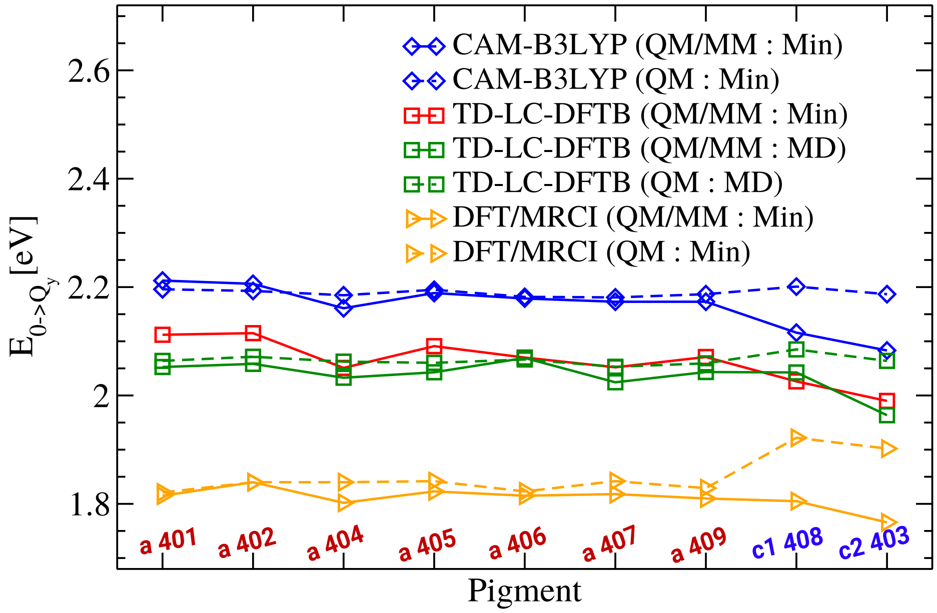

Figure S7: Excitation energy ladders for the FCP complex of *P. tricornutum* including (solid) and excluding (dashed) the QM/MM coupling to the protein environment in the excited state calculations. For all quantum chemistry approaches studied, there is a significant shift of the excitation energies of the Chl-c molecules.

Fig. S7, the excitation energies for most of the Chl molecules are blue-shifted when MM point charges are not taken into account. Moreover, for a few Chl pigments, the excitation energies are higher for the QM/MM-minimized structure compared to the average excitation energies along the 1 ns QM/MM MD simulation. As discussed in the main part, due to the averaging along a trajectory, the time-averaged data should be more reliable than that

based on a single conformation (including problems to really achieve a global minimum for the QM/MM-optimized structure). However, all the calculations presented here display the same trend in which the Chl-c molecules have the large energy shifts with that of Chl-c2 showing the largest one. This finding is due to the sensitivity of the Chl-c molecules to external electric fields created by the protein environment which affects the density difference between the ground and the Q_y excited states, leading to a rather unexpectedly large energy shift as explained in the main part. Since Chl-a molecules are least affected by the external electric fields as explained in the main text, their excitation energies are, however, not so drastically affected by the electrostatic environment for all the quantum-chemical methods as shown in Fig. S7.

Table S1: Q_y excitation energies (in eV) of the Chl molecules based on different QM methods on top of the QM/MM-optimized geometries. The average excitation energies based on the TD-LC-DFTB calculations along the QM/MM MD trajectory are given in brackets.

	CAM-B3LYP	ωB97X	TD-LC-DFTB	DFT/MRCI	ZINDO/S
Chl-a 401	2.212	2.221	2.112 (2.053)	1.815	1.859
Chl-a 402	2.206	2.197	2.115 (2.058)	1.840	1.854
Chl-a 404	2.161	2.142	2.051 (2.033)	1.802	1.786
Chl-a 405	2.189	2.175	2.091 (2.043)	1.823	1.807
Chl-a 406	2.179	2.158	2.070 (2.069)	1.815	1.784
Chl-a 407	2.173	2.149	2.052 (2.024)	1.818	1.756
Chl-a 409	2.173	2.156	2.071 (2.043)	1.810	1.793
Chl-c1 408	2.116	2.105	2.026 (2.042)	1.805	1.662
Chl-c2 403	2.083	2.078	1.990 (1.964)	1.766	1.654

S6 Excitation Energies Distributions of Chl-a and Chl-c1/c2 Molecules in Organic Solvent

Chl-a, Chl-c1 and Chl-c2 molecules were solvated individually using diethyl ether and acetone molecules by employing the GROMACS simulation engine. The topology and parameters for these solvent molecules are based on GAFF force fields and were prepared using the ACPYPE program suite[38]. The simulation box was considered cubic with a box length of

5 Å in each direction. For such a box, the Chl-a pigment was solvated by 868 ether or 1422 acetone molecules, the Chl-c1 pigment by 872 ether or 1425 acetone molecules and the Chl-c2 pigment by 872 ether or 1427 acetone molecules. The same equilibration steps were

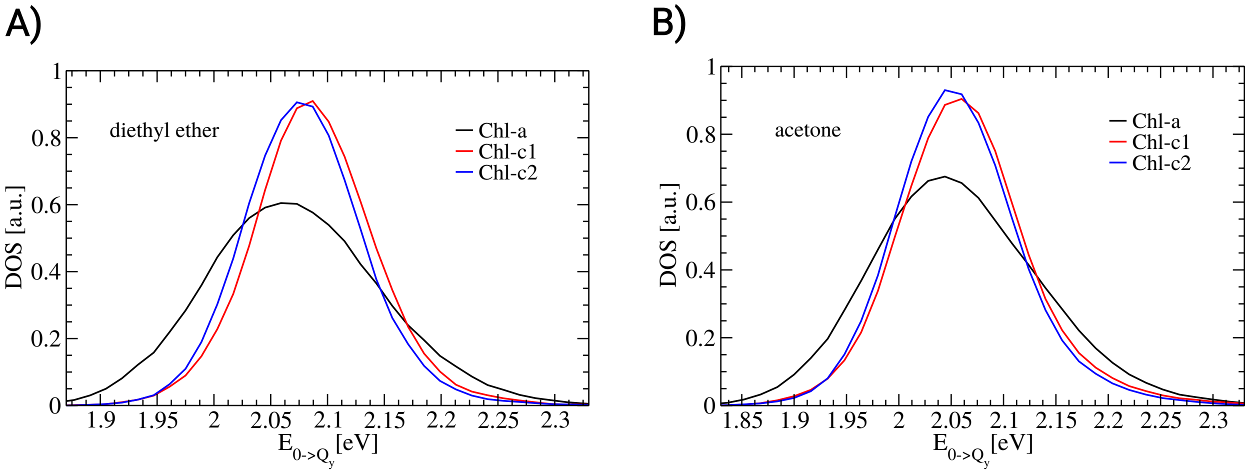

Figure S8: Excitation energy distributions of the Chl-a and Chl-c1/c2 pigments in the organic solvents A) diethyl ether and B) acetone based on a 1 ns-long QM/MM MD trajectory.

followed as for all other setups. First, an energy minimization was carried out, keeping a position restrain on the heaver atoms of the pigment molecules. Subsequently, a 2 ns NVT was performed followed by 5 ns NPT maintaining the same position restrain. Finally, a 20 ns NPT was performed without any position restrain. The integration time step was chosen to be 1 fs in each step. In a next step, we used the classically equilibrated structure for a 1.1 ns-long QM/MM MD simulation where for the last 1 ns trajectory snapshots were stored every 100 fs. This scheme produces 10,000 frames which were then utilized for excitation energy calculations based on TD-LC-DFTB method in a QM/MM fashion. Moreover, we followed the same setup for the excitation energy calculations as explained in the main text.

Fig. S8 displays the Q_y excitation energy distributions of the Chl-a and the Chl-c1/c2 pigments in both solvents. As one can see in Fig. S8 and Table 1 in the main text, in case of the solvent ether, the energies of the Chl-c molecule are significantly blue-shifted whereas in acetone the shift is less. This is because acetone is a more polar solvent than ether and thus, the pigment molecules can experience a more specific electric field due to a more specific first

and second solvation shell. Overall, the blue-shift in ether or acetone solvents is not as large as the experimental value listed in Table 1 in the main part. This shortcoming is likely due to the fact that liquid molecules moved a lot during the simulation, as compared to a roughly static environment in protein. Thus, one has to average over many configurations (along a trajectory) to obtain a decent sampling to really average out effects of small directed electric fields in individual configurations. In the case of proteins, however, the situation is simpler since there the Chl molecules are (more or less) fixed in space and incomplete sampling is not a problem. While for the high-level DFT/MRCI approach in gas phase, the blue-shift was well reproduced as can be seen in Table 1 in the main part, a usage of this numerical expensive scheme along a trajectory is not feasible and thus the TD-LC-DFTB approach was employed which adds uncertainties in the accuracy of the excitation energies. In addition, we would like to mention that within the FCP complex (with and without taking the protein environment into account in the excited state calculations) there is a significant shift, as can be seen in the subsequent section.

S7 Local Protein Environment around the Chl-c Molecules

In order to visualize the local protein environment of the Chl-c molecules, we have analyzed the amino acid residues in a 15 Å radius around the respective Mg atoms. It was found that GLN 143 and HIS 39 have strong non-covalent interactions with the Mg atoms of the respective pigments, i.e., Chl-c1 408 and Chl-c2 403 (see Fig. S9). These two residues are anchoring the Chl-c molecules within the protein matrix of the FCP complex during the dynamical simulations.

Moreover, we have calculated the distances between the amino acid residues GLN 143 and HIS 39 and the respective Mg atoms of the Chl-c pigments along the QM/MM MD trajectory. The distance distributions are shown in Fig. S9. In case of the distance between the oxygen atom of residue 143 and the respective magnesium atom, i.e., O(GLN 143)-Mg(Chl-c1 408),

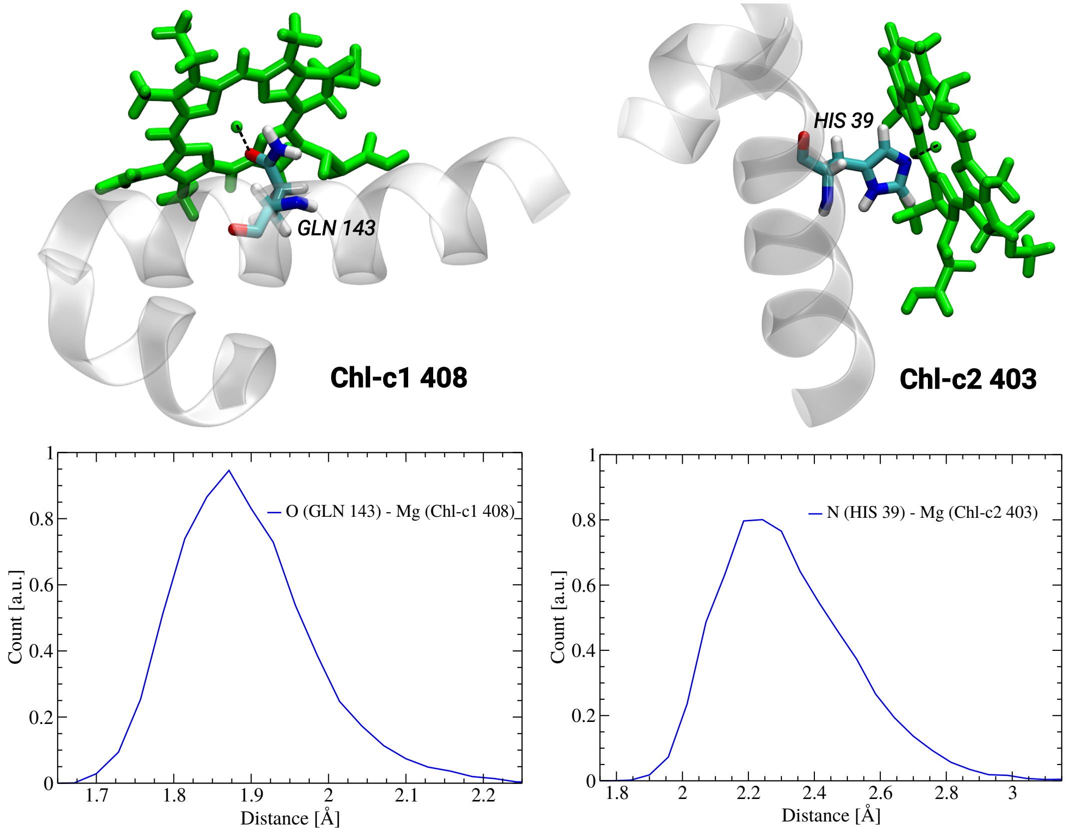

Figure S9: The upper panels show the local protein environments anchoring the pigments Chl-c1 408 and Chl-c2 403. Depicted in the lower panels are the distance distributions between the key atoms of the anchoring residues, i.e., oxygen and nitrogen, and the Mg atoms of Chl-c pigments along the QM/MM MD trajectory.

the distance fluctuates between 1.7-2.2 Å whereas in case of N(HIS 39)-Mg(Chl-c2 403), it lies within the range of 1.8 to 3 Å. These values suggest a strong binding which keeps the Chl-c molecules firmly coupled to these residues during the MD simulation. Furthermore, we have investigated the stability of the Chl-c pigments by calculating their RMSD values along the MD trajectory. In addition, the RMSD values of the C-α atoms of the nearby amino residues within a 15 Å radius around the Mg atoms are shown in Fig. S10 in order to check for major conformational changes of the protein environment. The RMSD curves reveal that the C-α atoms have very similar RMSD values for the nearby amino acid residues of the Chl-c1 and Chl-c2 pigments, whereas the Chl-c2 and especially the Chl-c1 pigments have higher values. Nevertheless, the small absolute magnitudes of these RMSD values indicate that the Chl-c pigments and their nearby protein environments are highly stable during the simulations.

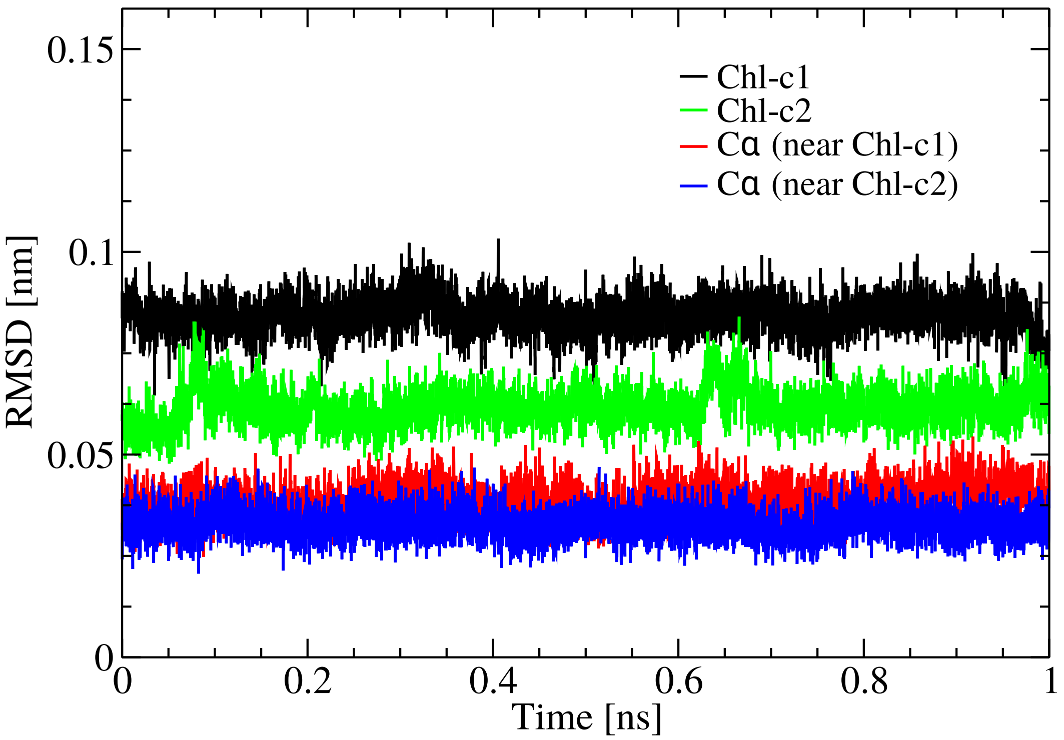

Figure S10: RMSD values of the Chl-c molecules together with the C-α RMSD values of the nearby amino acid residues along the QM/MM MD trajectory.

This test rules out artifacts in the excitation energies due to major conformational change during the QM/MM MD simulation.

S8 Difference Densities for all Chl-a and Chl-c Molecules with and without QM/MM Coupling

The electric field can affect the highest occupied molecular orbital (HOMO) and lowest unoccupied molecular orbital (LUMO) in a particular electronic transition. Thus, it might be a good idea to have a closer look at a pictorial representation of the electron density delocalization of these orbitals. However, because of the complex electronic structure of the Chl molecules, many HOMOs and LUMOs can participate in the Q_y excited state within a TD-DFT framework. For this reason, we have extracted the density difference between these HOMOs and LUMOs for the QM/MM-minimized geometries with and without QM/MM

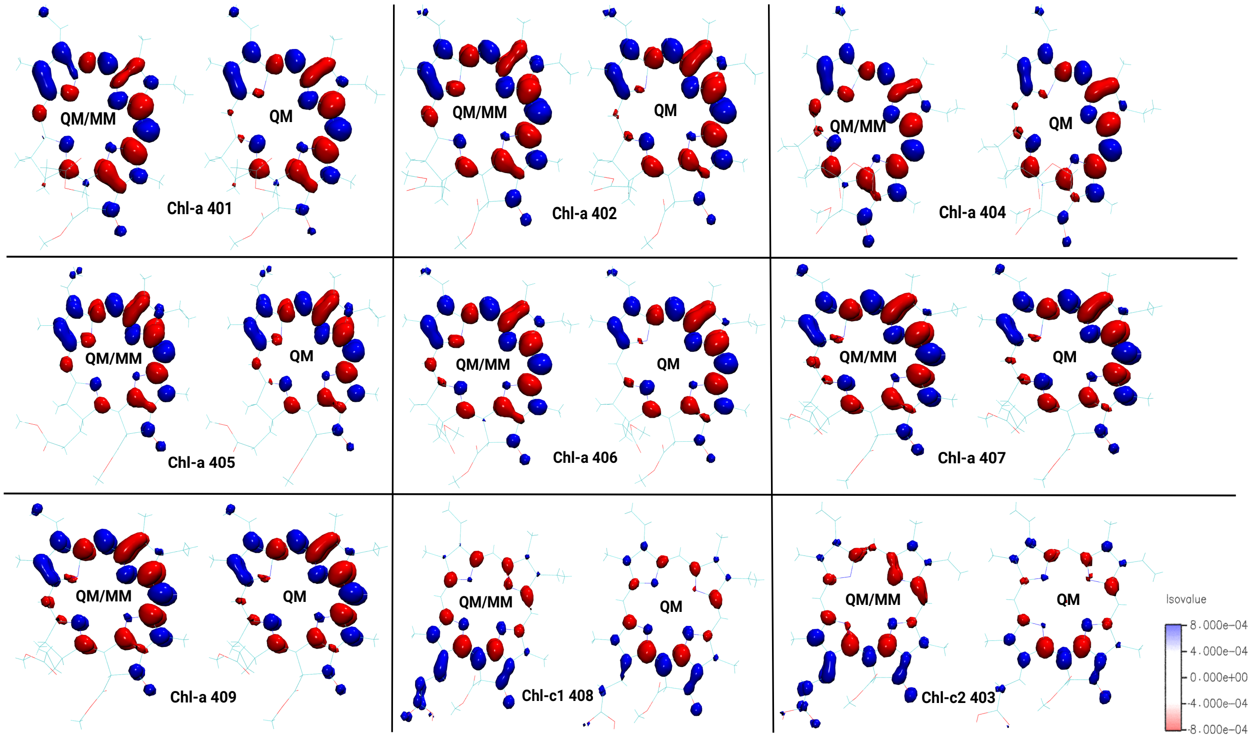

Figure S11: Same as shown in Fig. 2B of the main text, but for all pigment molecules.

coupling. The CAM/B3LYP functional together with the Def2-TZVP basis set has been utilized for the TD-DFT calculations as implement in ORCA [39] and the extracted difference densities have been visualized via the VMD package [28] and shown in Fig. S11. Only the Chl-c pigments show a large change in the difference densities with Chl-c2 displaying the largest effect. This finding again highlights the strong effect of the electric field on these pigments, reflected by the peculiar large energy shift at all studied levels of quantum-chemical calculations as shown earlier.

S9 Effect of External Electric Field on the Chl-a and Chl-c Molecules

In the main part, the effect of a homogeneous electric field on the excitation energies and density difference along the directions x, y, and z are shown and explained. Here, similar

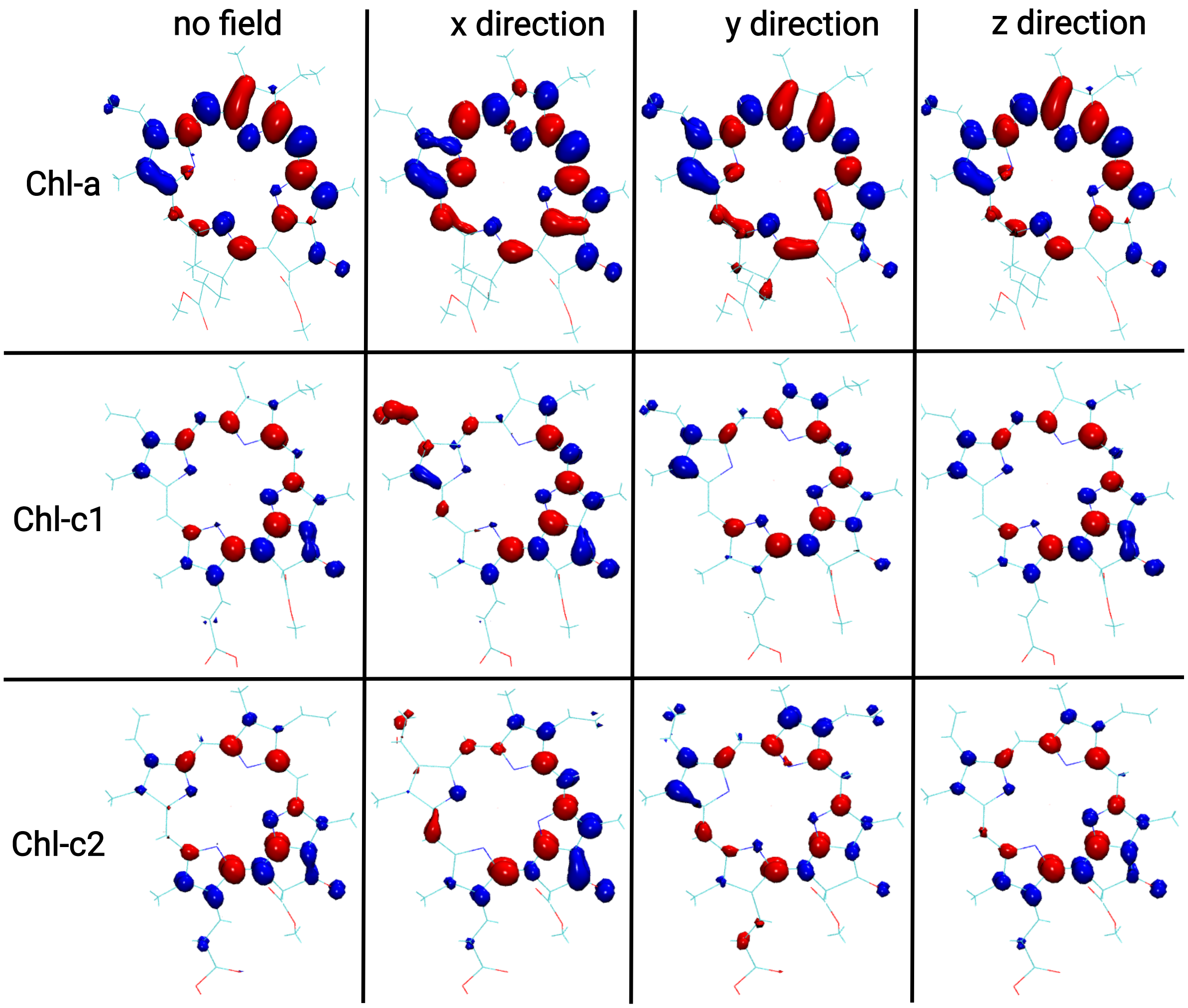

Figure S12: Same as shown in Fig. 3 of the main text, but for the opposite field directions.

calculations have repeated for the opposite directions of those electric fields. Excitation energies are tabulated in the Table 2 in the main text while the associated density differences are depicted in Fig. S12. As one can see, the negative component of electric field does not significantly change the excitation energy as well as the density difference for the Chl-a or Chl-c molecules. Most importantly, fields in the negative y direction has no significant effect on the Chl-c molecules as we have found for its positive counterpart as shown in the main text.

Moreover, we have extracted the transition dipole moments from the Chl-c molecules for gas-phase conformations as well as in the presence of homogeneous electric fields as shown in Table S2. As can be seen, an electric field in the $+y$ direction significantly changes

Table S2: Q_y transition moment vectors (x, y and z in atomic units) and their absolute values ($|\mu|^2$) for the Chl-c molecules based on a gas phase-optimized conformation as well as in presence of homogeneous electric fields in directions as indicated and described in the text. The y-component and the absolute dipole moments in presence of an electric field in $+y$ direction are highlighted in bold.

	x		y		z		$\|\mu\|^2$	
	Chl-c1	Chl-c2	Chl-c1	Chl-c2	Chl-c1	Chl-c2	Chl-c1	Chl-c2
Gas-phase	-0.97877	0.66742	-0.16851	0.33786	-0.05820	0.08770	0.98978	0.56729
$+x$ direction	-1.05172	-0.23812	0.18972	-0.66385	0.04965	-0.14646	1.14458	0.51884
$+y$ direction	1.02708	0.41106	**1.67363**	**1.51813**	0.21795	0.30740	**3.90342**	**2.56819**
$+z$ direction	0.91063	-0.76532	0.29412	-0.48529	0.05792	-0.10970	0.91910	0.83325
$-x$ direction	-1.13669	0.63668	0.35227	-0.33291	-0.11145	-0.04759	1.42858	0.51845
$-y$ direction	-0.78395	-0.47170	-0.17039	-0.42013	0.00442	-0.07748	0.64364	-0.07748
$-z$ direction	1.05176	-0.57142	0.06339	-0.20585	0.05865	-0.06799	1.11367	0.37352

the y-component and the absolute dipole moment of the Chl-c1/c2 molecules. This finding again supports the electric field sensitivity of the Chl-c molecules and therefore the observed environmental effects in the FCP complex.

S10 Excitonic Couplings and the Exciton Hamiltonian

Within the tight-binding approximation and the single-exciton manifold, the system exciton Hamiltonian is given by

$$H_S = \sum_m E_m |m\rangle \langle m| + \sum_{n \neq m} V_{mn} |n\rangle \langle m| \ , \tag{1}$$

where E_m refers to the local excitation energy, i.e., the site energy, of pigment m and V_{mm} denotes the excitonic coupling between pigments m and n. In the present study, the time-averaged system Hamiltonian is constructed based on the Q_y excitation energies from the 1 ns-long QM/MM MD trajectory as shown in Fig. 4 of the main text and the excitonic coupling are based on the 1 μs-long classical MD trajectory as explained below. The resulting system Hamiltonian is detailed in Table S2. At this point, a comment is in order concerning the connection between the Q_y excitation energies and the site energies for chromophores

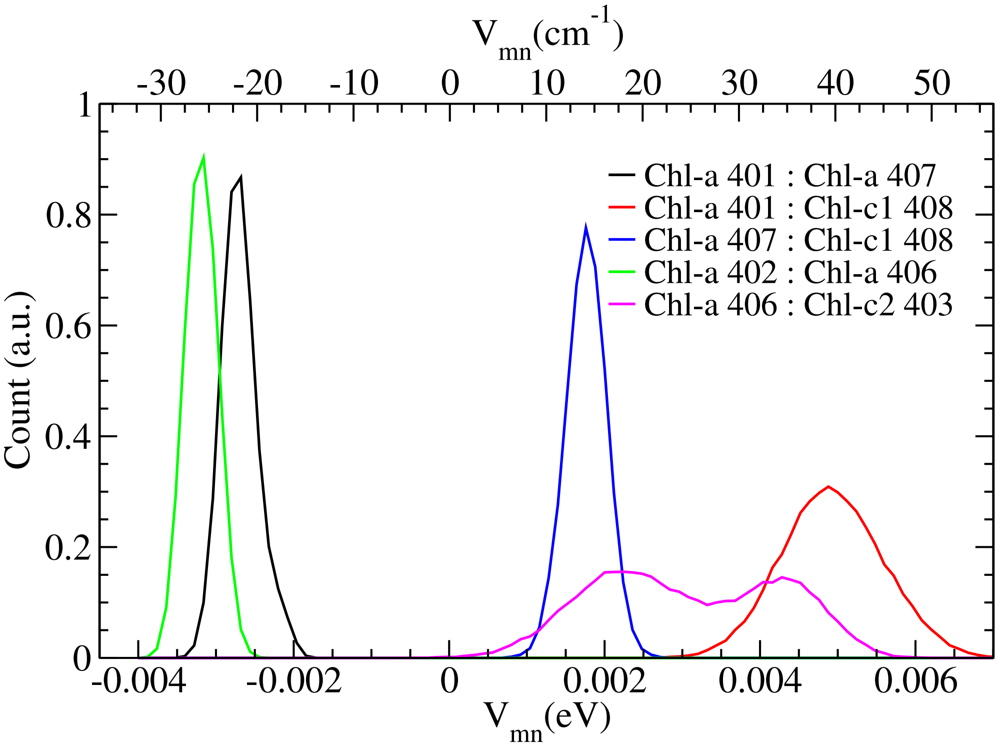

Figure S13: Excitonic coupling values between those pigment pairs whose average absolute coupling values are larger than 10 cm^{-1}.

in a protein and solvent environment. Here, we assume that the reorganization timescale is significantly longer than the transfer time of excitons to neighboring pigments. If all pigments are of the same kind and in similar environments, less of an issue exists to identify the site energies in a tight-binding model with the Q_y excitation energies. In the present case, we have to handle two different pigment types with fairly different spectral densities. In such a case, a more detailed treatment of the intra- and intermolecular spectral densities and reorganization energies might be necessary to gain a very accurate model. A non-adiabatic treatment would certainly be beneficial but is clearly beyond the scope of this study, but work in this direction is ongoing.

As just mentioned, the excitonic couplings are needed to construct the system Hamiltonian. To this end, we have employed the so-called TrESP (transition charges from electrostatic potential) approach proposed by Renger and co-workers[40,41]. In this approach, the

coupling values are calculated using

$$V_{mn}^{\text{TrESP}} = \frac{f}{4\pi\epsilon_0} \sum_{I,J}^{m,n} \frac{q_I^T \cdot q_J^T}{\mid r_m^I - r_n^J \mid} \tag{2}$$

where q_I^T and q_J^T are the atomic transition charge present at atoms I and J of pigments m and n, respectively. The transition charges have been calculated at the TD-DFT CAM-B3LYP/def2-TZVP level of theory with the help of the ORCA[39] and Multiwfn[27] programs as explained in Ref. 42. For the Chl-a molecules, the transition charges taken from Ref. 42, whereas for the Chl-c1 and Chl-c2 molecules, they have been calculated in the present study and are tabulated in Table S4 at the end of this SI. Moreover, the charges have been rescaled to reproduce the experimental transition dipole moments. In case of Chl-a, this rescaling factor was 0.81 as considered in Ref. 42. Since no experimental transition dipole moments have been reported for Chl-c molecules, we have considered 0.81 scaling factor for Chl-c transition charges as well. Furthermore, we have employed a constant screening factor of 0.69 that is often utilized for the LH complexes of plants in order to account for the environmental influences on the excitonic couplings[43]. Here, we would like to point that since the Q_y transition dipole moments of the Chl-c molecules are very sensitive to the environmental electric field, especially along the $+y$ direction as shown in the Table S2, and in order to properly account for polarization effects on the excitonic couplings, one needs to perform "on-the-fly" calculations of the couplings including environmental point charges[44] which is beyond the scope of the present investigation. A 1 μs-long classical MD trajectory was utilized for the coupling calculations, as described in the main text.

The excitonic couplings in the studied FCP complex are very small in magnitude, and the coupling value distributions of those pigment pairs whose average absolute values are larger than 10 cm^{-1} are shown in Fig. S13. The time-averaged coupling and site energies from the 1 ns-ling QM/MM MD trajectory are put together to built the time-averaged system Hamiltonian, as given in Table S3. This system Hamiltonian has been used for modeling of

Table S3: Time-averaged system exciton Hamiltonian matrix of the studied FCP complex based on the excitonic coupling values along 1 μs classical MD trajectory and site energies from 1 ns QM/MM MD simulation. The site energies and couplings (in cm^{-1}) with absolute values above 10 cm^{-1} are shown in boldface.

	a 401	a 402	a 404	a 405	a 406	a 407	a 409	c1 408	c2 403
a 401	**16555**	-3.12	-2.89	1.10	3.95	**-22.19**	-3.73	**39.30**	0.46
a 402	-3.12	**16602**	5.12	-0.94	**-26.19**	-8.84	-2.48	5.12	7.72
a 404	-2.89	5.12	**16395**	0.82	-4.73	-2.07	0.43	0.27	-4.51
a 405	1.10	-0.94	0.82	**16476**	3.24	-2.25	-0.73	-0.80	1.20
a 406	3.95	**-26.19**	-4.74	3.24	**16690**	-0.42	-1.70	0.38	**24.34**
a 407	**-22.19**	-8.84	-2.07	-2.25	-0.42	**16327**	3.90	**13.74**	0.99
a 409	-3.73	-2.48	0.43	-0.73	-1.70	3.90	**16480**	-1.53	-0.33
c1 408	**39.30**	5.12	0.27	-0.80	0.38	**13.74**	-1.53	**16474**	-0.67
c2 403	0.46	7.72	-4.51	1.20	**24.34**	0.99	-0.33	-0.67	**15837**

the absorption spectra, as detailed in the main text.

S11 Spectral Densities of Individual Pigments

An important quantity determining the form of the absorption profile is the spectral density, which mainly represent the frequency-dependent coupling between the system and its environment. It has been extracted from the site energies fluctuations based on the 60 ps-long QM/MM MD trajectory as explained in the **Materials and Methods** section. Recently, we have developed a multiscale scheme which is robust and rather accurate in determining spectral densities. This scheme has been employed to various bacterial and plant LH systems and the results are in good agreement with the experimental counterparts[7,8,42,45]. In the present study, we have followed the same strategy in order to obtain the spectral densities of the individual Chl-a and Chl-c pigments. Details of the approach which has been reviewed recently[46] can be found elsewhere[7,8,42,45].

In addition to the system Hamiltonian and spectral densities, we have extracted the time-averaged transition dipole moments from the 1 ns-long QM/MM MD trajectory which also served as an input for the calculation of the absorption spectrum at a temperature of 300 K based on the FCE method as developed by Cao and co-workers[47].

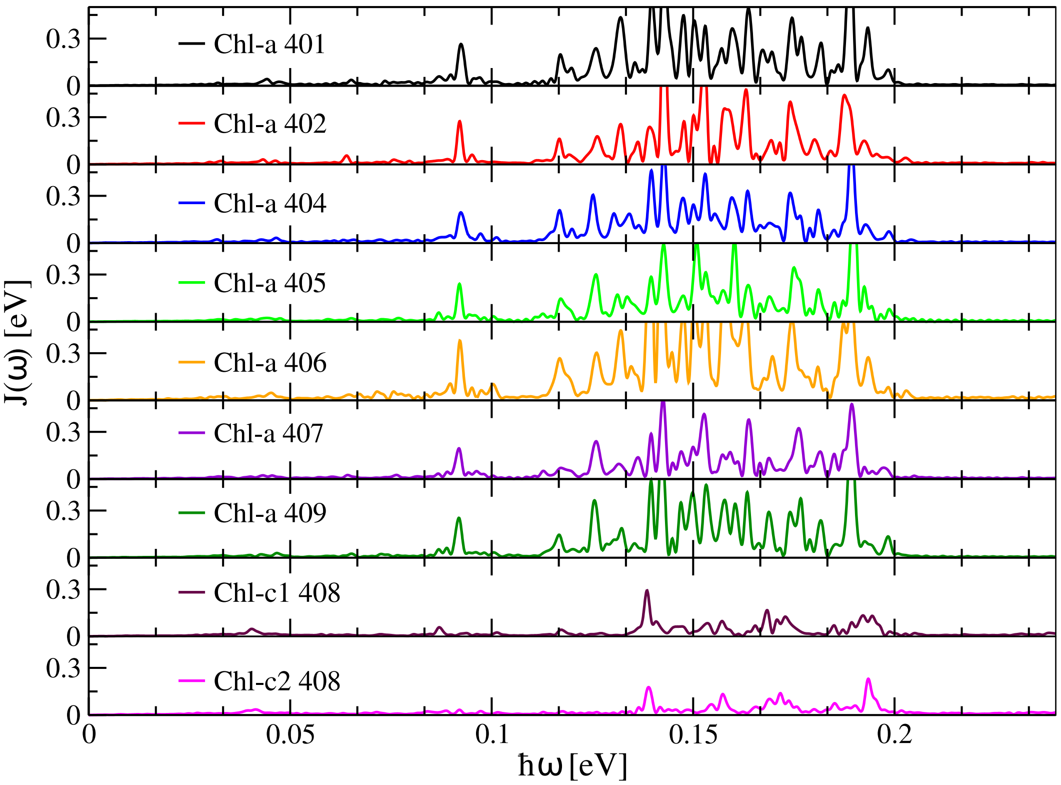

Figure S14: Spectral densities of the individual Chl-a and Chl-c pigments in the FCP complex.

The average spectral densities of the Chl-a and Chl-c molecules of the studied FCP complex are depicted in Fig. S15 whereas the spectral densities for the individual pigments are provided in Fig. S14. Although, there is no experimental spectral density available for any FCP complex so far, the Chl-a molecules show spectral densities similar to those of plant LH complexes[8,42,45,46]. As an example, we have compared in Fig. S15 the computed spectral density of the Chl-a molecules in the FCP protein with the experimental one from the major light-harvesting complex of plant photosystem, i.e., LHCII[48]. Although the average spectral density of the Chl-a molecules does not perfectly match the experimental one, overall both results show a nice agreement taking into account the size of the complexes. In the case of the Chl-c pigments, the low-frequency regions are quite similar to those of the Chl-a molecules, the high-frequency regions differ significantly in terms of the number of peaks and their intensities.

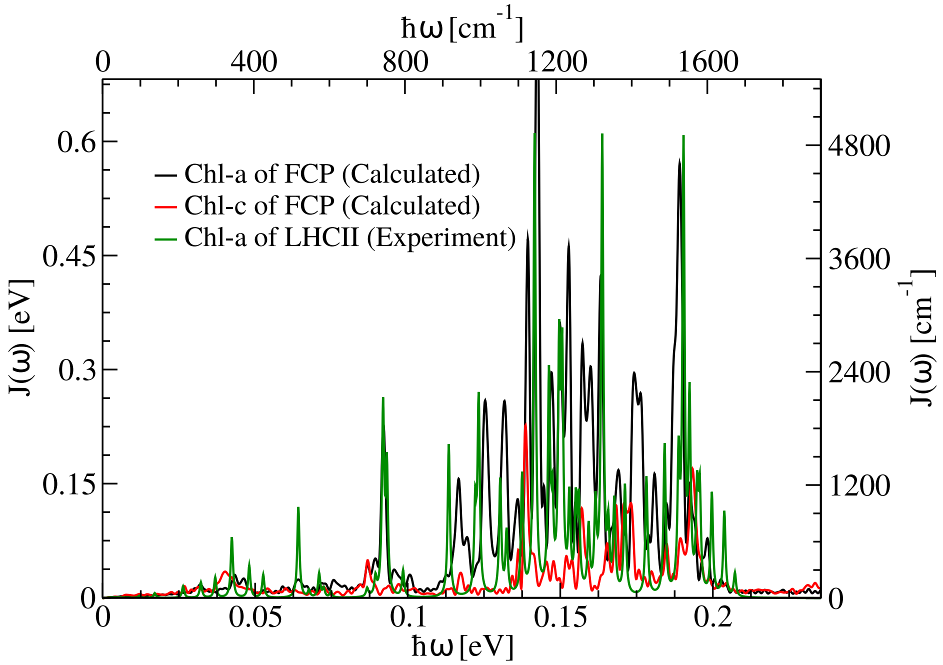

Figure S15: Average spectral densities of the Chl-a and Chl-c pigment molecules in the studied FCP complex in comparison with the experimental spectral density of the LHCII complex[48].

The spectral densities of the individual pigments have been calculated from the site energies fluctuations based on the 60 ps-long QM/MM MD trajectory as explained in the main text. The average spectral density of the Chl-a and Chl-c molecules are given in the main text, while the individual ones are presented in Fig. S14. For the Chl-a molecules, one obtains similar profiles for the spectral densities as found earlier for plant LH complexes[8,42,45]. For the Chl-c molecules, however, the spectral densities contain a lower number of peaks as well as lower amplitudes in the high-frequency region. The high-frequency peaks are mainly due to intramolecular vibrational modes arising due to vibrations involving the C=C, C=N, C=O bonds in the Mg-porphyrin ring. Hence, the differences in the high-frequency region between Chl-a and Chl-c molecules are probably due to the fact that the Mg-porphyrin ring of Chl-c molecules are more rigid compare to those of the Chl-a molecules as described in the main text. The individual spectral densities have been utilized as input parameters during the modeling of the absorption spectra with the full second-order cumulant expansion (FCE) method[47] as shown in the main text.

S12 Absorption Spectra

In this section, we report on absorption spectra based on the calculated spectral densities. Again, we performed the calculation for the full spectral and with one where only the low-frequency part until 800 cm^{-1} is taken into account. The results are depicted in Fig. S16. Here, the computed spectrum is shifted by about -726 cm^{-1} and -1452 cm^{-1} with respect to the experimental results for the full and the low-frequency spectral densities. As one can see,

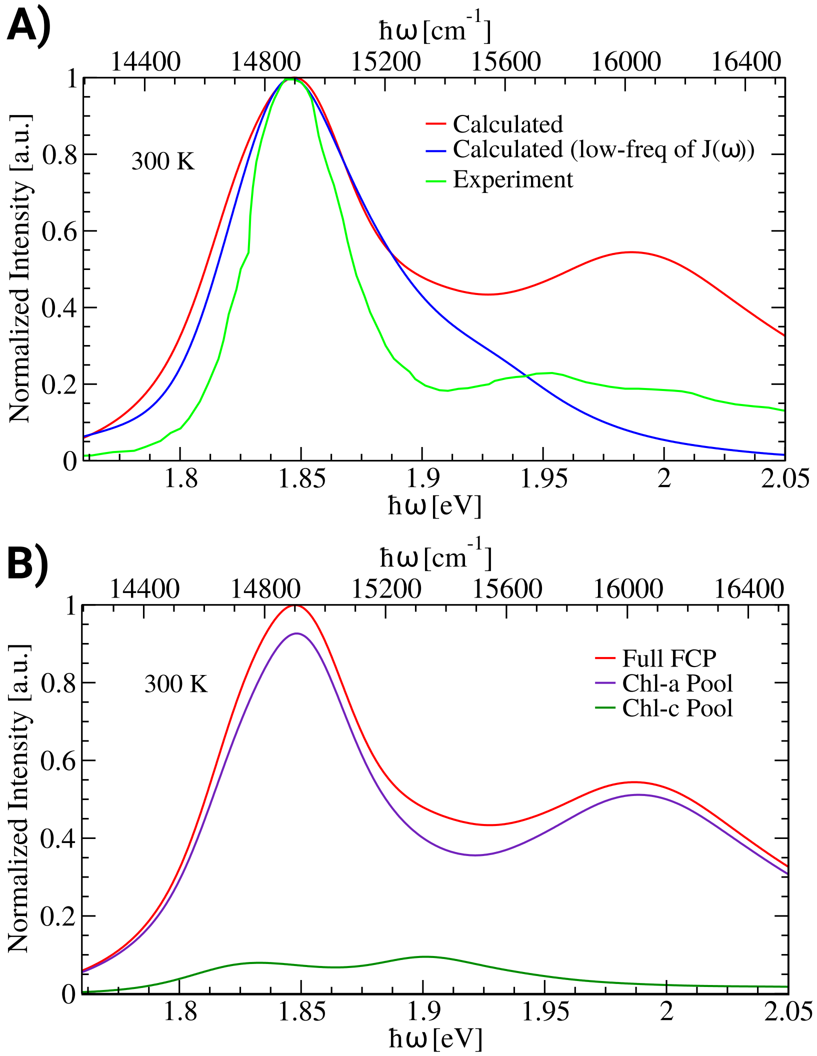

Figure S16: Same as Fig. 4 in the main text, but based on the atomistic spectral densities.

the overall line-shape of the spectrum is similar to the one obtained using the experimental spectral density. The high-energy side band, however, is significantly higher in amplitude

in the one based on the full atomistic spectral density. Thus, the difference in the high-frequency peaks in the calculated and experimental spectral densities lead to a quite clear difference in the side band of the absorption spectrum (see Fig. 4 in the main text). At the same time, Fig. S16 reveals that the Chl-c molecules contribute only very little to the overall line-shape of the total absorption spectrum of the FCP complex as already seen in Fig. 4 in the main part based on the experimental spectral density. Hence, it is clear that the high-energy side band is due to the vibrational progression of the Chl-a molecules rather than due to the Q_y peak of the Chl-c irrespective of the choice of spectral density.

Table S4: Transition charges (in atomic unit) of the heavy atoms of the Chl-a and Chl-c pigment molecules. The atom names are based on the crystal structure of the studied FCP complex[49]. The charges of hydrogen atoms were set to zero during the fitting procedure. These charges are usually rescaled to reproduce experimental dipole moments, as explained earlier. The Chl-a transition charges are extracted from the Ref. 42 whereas for Chl-c1/c2 are derived in the present study. The Q_y transition dipole moment of Chl-a is assigned approximately along NB-ND axis, while for Chl-c1/c2 they are assigned approximate along NA-ND axis as shown in Fig. S2.

**Chl-a**	**Charge $\times$ 10^3**	**Chl-c1**	**Charge $\times$ 10^3**	**Chl-c2**	**Charge $\times$ 10^3**
MG	5.906	NB	75.296	NB	68.655
CHA	-112.870	ND	-83.977	ND	-76.107
CHB	42.984	C1A	61.214	C1A	46.952
CHC	133.029	C1B	-114.655	C1B	-120.061
CHD	-61.417	C1C	-76.78	C1C	-49.131
NA	-17.786	C1D	116.952	C1D	122.968
C1A	119.324	C2A	10.913	C2A	-11.489
C2A	-0.944	C2B	2.089	C2B	6.132
C3A	-2.051	C2C	10.54	C2C	8.03
C4A	-98.329	C2D	11.683	C2D	4.981
CMA	-6.256	C3A	9.415	C3A	6.055
CAA	0.598	C3B	-5.619	C3B	-18.07

CBA	2.377	C3C	-11.526	C3C	-4.411
CGA	-0.820	C3D	17.495	C3D	15.842
O1A	-2.916	C4A	-128.398	C4A	-130.105
O2A	5.314	C4B	-59.077	C4B	-22.195
NB	81.451	C4C	120.982	C4C	125.702
C1B	-77.909	C4D	48.435	C4D	31.36
C2B	-1.300	CAA	-4.445	CAA	-2.156
C3B	-1.977	CAB	-3.486	CAB	7.779
C4B	-131.939	CAC	1.082	CAC	-5.057
CMB	-20.197	CAD	-7.792	CAD	-1.429
CAB	-0.882	CBA	-8.162	CBA	-13.837
CBB	-26.859	CBB	-20.429	CBB	-20.39
NC	21.980	CBC	-0.362	CBC	1.389
C1C	-128.399	CBD	19.442	CBD	13.515
C2C	14.758	CED	2.245	CED	2.068
C3C	-14.975	CGA	-1.284	CGA	-7.41
C4C	63.002	CMC	13.053	CGD	4.388
CMC	1.853	CGD	-85.305	CHA	-62.325
CAC	-4.869	CHA	147.368	CHB	160.83
CBC	-2.990	CHB	85.936	CHC	56.007
ND	-84.710	CHC	-131.521	CHD	-144.483
C1D	89.460	CHD	-4.163	CMA	-6.966
C2D	15.705	CMA	-7.797	CMB	-5.537
C3D	14.556	CMB	6.534	CMC	8.975
C4D	109.052	CMC	16.984	CMD	13.46
CMD	27.878	CMD	19.42	NA	30.726
CAD	1.074	NA	-21.769	NC	-39.582

OBD	21.169	NC	-4.561	O1A	-5.313
CBD	28.287	O1A	-3.962	O1D	-0.87
CGD	-5.231	O1D	-2.199	O2A	-2.456
O1D	6.431	O2D	-11.56	O2D	-6.113
O2D	-7.232	OBD	15.697	OBD	13.295
CED	7.038	MG	7.884	MG	6.385
C1	-0.370				

References

(1) Chrysafoudi, A.; Maity, S.; Kleinekathöfer, U.; Daskalakis, V. Robust Strategy for Photoprotection in the Light-Harvesting Antenna of Diatoms: A Molecular Dynamics Study. *J. Phys. Chem. Lett.* **2021**, *12*, 9626–9633.

(2) Gaus, M.; Cui, Q.; Elstner, M. DFTB3: Extension of the Self-Consistent-Charge Density-Functional Tight-Binding Method (SCC-DFTB). *J. Chem. Theory Comput.* **2011**, *7*, 931–948.

(3) Gaus, M.; Goez, A.; Elstner, M. Parametrization and Benchmark of DFTB3 for Organic Molecules. *J. Chem. Theory Comput.* **2013**, *9*, 338–354.

(4) Duan, Y.; Wu, C.; Chowdhury, S.; Lee, M. C.; Xiong, G.; Zhang, W.; Yang, R.; Cieplak, P.; Luo, R.; Lee, T.; Caldwell, J.; Wang, J.; Kollman, P. A Point-Charge Force Field for Molecular Mechanics Simulations of Proteins Based on Condensed-Phase Quantum Mechanical Calculations. *J. Comput. Chem.* **2003**, *24*, 1999–2012.

(5) Kubař, T.; Welke, K.; Groenhof, G. New QM/MM Implementation of the DFTB3 Method in the Gromacs Package. *J. Comput. Chem.* **2015**, *36*, 1978–1989.

(6) Bold, B. M.; Sokolov, M.; Maity, S.; Wanko, M.; Dohmen, P. M.; Kranz, J. J.; Kleinekathöfer, U.; Höfener, S.; Elstner, M. Benchmark and Performance of Long-Range Corrected Time-Dependent Density Functional Tight Binding (LC-TD-DFTB) on Rhodopsins and Light-Harvesting Complexes. *Phys. Chem. Chem. Phys.* **2020**, *22*, 10500–10518.

(7) Maity, S.; Bold, B. M.; Prajapati, J. D.; Sokolov, M.; Kubař, T.; Elstner, M.; Kleinekathöfer, U. DFTB/MM Molecular Dynamics Simulations of the FMO Light-Harvesting Complex. *J. Phys. Chem. Lett.* **2020**, *11*, 8660–8667.

(8) Maity, S.; Daskalakis, V.; Elstner, M.; Kleinekathöfer, U. Multiscale QM/MM Molecular Dynamics Simulations of the Trimeric Major Light-Harvesting Complex II. *Phys. Chem. Chem. Phys.* **2021**, *23*, 7407–7417.

(9) Kranz, J. J.; Elstner, M.; Aradi, B.; Frauenheim, T.; Lutsker, V.; Garcia, A. D.; Niehaus, T. A. Time-Dependent Extension of the Long-Range Corrected Density Functional Based Tight-Binding Method. *J. Chem. Theory Comput.* **2017**, *13*, 1737–1747.

(10) Yanai, T.; Tew, D. P.; Handy, N. C. A New Hybrid Exchange–Correlation Functional Using the Coulomb-Attenuating Method (CAM-B3LYP). *Chem. Phys. Lett.* **2004**, *393*, 51–57.

(11) Chai, J.-D.; Head-Gordon, M. Long-Range Corrected Hybrid Density Functionals With Damped Atom-Atom Dispersion Corrections. *Phys. Chem. Chem. Phys.* **2008**, *10*, 6615–6620.

(12) Marian, C. M.; Heil, A.; Kleinschmidt, M. The DFT/MRCI Method. *WIREs Comput Mol Sci* **2018**, *0*, e1394.

(13) Hirata, S.; Head-Gordon, M. Time-dependent Density Functional Theory within the Tamm–Dancoff Approximation. *Chem. Phys. Lett.* **1999**, *314*, 291–299.

(14) Neese, F.; Wennmohs, F.; Hansen, A.; Becker, U. Efficient, Approximate and Parallel Hartree-Fock and Hybrid DFT Calculations. A 'Chain-of-Spheres' Algorithm for the Hartree-Fock Exchange. *Chem. Phys.* **2009**, *356*, 98–109.

(15) Neese, F.; Wennmohs, F.; Becker, U.; Riplinger, C. The ORCA Quantum Chemistry Program Package. *J. Chem. Phys.* **2020**, *152*, 224108.

(16) Hourahine, B.; Aradi, B.; Blum, V.; Bonafé, F.; Buccheri, A.; Camacho, C.; Cevallos, C.; Deshaye, M. Y.; Dumitrică, T.; Dominguez, A.; Ehlert, S.; Elstner, M.; Van Der Heide, T.; Hermann, J.; Irle, S.; Kranz, J. J.; Köhler, C.; Kowalczyk, T.; Kubař, T.; Lee, I. S.; Lutsker, V.; Maurer, R. J.; Min, S. K.; Mitchell, I.; Negre, C.; Niehaus, T. A.; Niklasson, A. M. N.; Page, A. J.; Pecchia, A.; Penazzi, G.; Persson, M. P.; Řezáč, J.; Sánchez, C. G.; Sternberg, M.; Stöhr, M.; Stuckenberg, F.; Tkatchenko, A.; Yu, V. W.-Z.; Frauenheim, T. DFTB+, a Software Package for Efficient Approximate Density Functional Theory Based Atomistic Simulations. *J. Chem. Phys.* **2020**, *152*, 124101.

(17) Maity, S.; Gelessus, A.; Daskalakis, V.; Kleinekathöfer, U. On a Chlorophyll-Caroteinoid Coupling in LHCII. *Chem. Phys.* **2019**, *526*, 110439.

(18) Olbrich, C.; Kleinekathöfer, U. Time-Dependent Atomistic View on the Electronic Relaxation in Light-Harvesting System II. *J. Phys. Chem. B* **2010**, *114*, 12427–12437.

(19) Jansen, T. L. C.; Knoester, J. Nonadiabatic Effects in the Two-dimensional Infrared Spectra of Peptides: Alanine Dipeptide. *J. Phys. Chem. B* **2006**, *110*, 22910–22916.

(20) Sardjan, A. S.; Westerman, F. P.; Ogilvie, J. P.; Jansen, T. L. Observation of Ultrafast Coherence Transfer and Degenerate States with Polarization-Controlled Two-Dimensional Electronic Spectroscopy. *J. Phys. Chem. B* **2020**, *124*, 9420–9427.

(21) Jansen, T. L. Computational Spectroscopy of Complex Systems. *J. Chem. Phys.* **2021**, *155*, 170901.

(22) Bondarenko, A. S.; Knoester, J.; Jansen, T. L. C. Comparison of Methods to Study Excitation Energy Transfer in Molecular Multichromophoric Systems. *Chem. Phys.* **2020**, *529*, 110478.

(23) Hamm, P.; Zanni, M. *Concepts and Methods of 2D Infrared Spectroscopy*; Cambridge University Press, 2011.

(24) Liang, C.; Jansen, T. L. C. An Efficient N3-Scaling Propagation Scheme for Simulating Two-Dimensional Infrared and Visible Spectra. *J. Chem. Theory Comput.* **2012**, *8*, 1706–1713.

(25) Songaila, E.; Augulis, R.; Gelzinis, A.; Butkus, V.; Gall, A.; Büchel, C.; Robert, B.; Zigmantas, D.; Abramavicius, D.; Valkunas, L. Ultrafast Energy Transfer from Chlorophyll c_2 to Chlorophyll a in Fucoxanthin–Chlorophyll Protein Complex. *J. Phys. Chem. Lett.* **2013**, *4*, 3590–3595.

(26) Gelzinis, A.; Augulis, R.; Büchel, C.; Robert, B.; Valkunas, L. Confronting FCP Structure with Ultrafast Spectroscopy Data: Evidence for Structural Variations. *Phys. Chem. Chem. Phys.* **2021**, *23*, 806–821.

(27) Lu, T.; Chen, F. Multiwfn: A Multifunctional Wavefunction Analyzer. *J. Comput. Chem.* **2012**, *33*, 580–592.

(28) Humphrey, W. F.; Dalke, A.; Schulten, K. VMD – Visual Molecular Dynamics. *J. Mol. Graph.* **1996**, *14*, 33–38.

(29) Shim, S.; Rebentrost, P.; Valleau, S.; Aspuru Guzik, A. Atomistic Study of the Long-Lived Quantum Coherences in the Fenna-Matthew-Olson Complex. *Biophys. J.* **2012**, *102*, 649–660.

(30) Grimme, S.; Waletzke, M. A Combination of Kohn-Sham Density Functional Theory and Multi-Reference Configuration Interaction Methods. *J. Chem. Phys.* **1999**, *111*, 5645–5655.

(31) Lyskov, I.; Kleinschmidt, M.; Marian, C. M. Redesign of the DFT/MRCI Hamiltonian. *J. Chem. Phys.* **2016**, *144*, 034104.

(32) Heil, A.; Marian, C. M. DFT/MRCI Hamiltonian for Odd and Even Numbers of Electrons. *J. Chem. Phys.* **2017**, *147*, 194104.

(33) Heil, A.; Kleinschmidt, M.; Marian, C. M. On the Performance of DFT/MRCI Hamiltonians for Electronic Excitations in Transition Metal Complexes: The Role of the Damping Function. *J. Chem. Phys.* **2018**, *149*, 164106.

(34) Ridley, J.; Zerner, M. C. An Intermediate Neglect of Differential Overlap Technique for Spectroscopy: Pyrrole and the Azines. *Theor. Chim. Acta* **1973**, *32*, 111–134.

(35) Thompson, M. A.; Zerner, M. C. A Theoretical Examination of the Electronic Structure and Spectroscopy of the Photosynthetic Reaction Center from Rhodopseudomonas Viridis. *J. Am. Chem. Soc.* **1991**, *113*, 8210–8215.

(36) Papagiannakis, E.; van Stokkum, I. H.; Fey, H.; Büchel, C.; van Grondelle, R. Spectroscopic Characterization of the Excitation Energy Transfer in the Fucoxanthin–Chlorophyll Protein of Diatoms. *Photosynth. Res.* **2005**, *86*, 241–250.

(37) Gelzinis, A.; Abramavicius, D.; Valkunas, L. Absorption Lineshapes of Molecular Aggregates Revisited. *J. Chem. Phys.* **2015**, *142*, 154107.

(38) Da Silva, A. W. S.; Vranken, W. F. ACPYPE-Antechamber Python Parser Interface. *BMC Res. Notes* **2012**, *5*, 367.

(39) Neese, F. Software Update: The ORCA Program System, Version 4.0. *WIREs Comput. Mol. Sci.* **2018**, *8*, e1327.

(40) Madjet, M. E.; Abdurahman, A.; Renger, T. Intermolecular Coulomb Couplings from Ab Initio Electrostatic Potentials: Application to Optical Transitions of Strongly Coupled Pigments in Photosynthetic Antennae and Reaction Centers. *J. Phys. Chem. B* **2006**, *110*, 17268–81.

(41) Renger, T.; Madjet, M.-A.; Schmidt am Busch, M.; Adolphs, J.; Müh, F. Structure-based Modeling of Energy Transfer in Photosynthesis. *Photosynth. Res.* **2013**, *116*, 367–388.

(42) Maity, S.; Sarngadharan, P.; Daskalakis, V.; Kleinekathöfer, U. Time-Dependent Atomistic Simulations of the CP29 Light-Harvesting Complex. *J. Chem. Phys.* **2021**, *155*, 055103.

(43) Renger, T.; Müh, F. Theory of Excitonic Couplings in Dielectric Media: Foundation of Poisson-TrEsp Method and Application to Photosystem I Trimers. *Photosynth. Res.* **2012**, *111*, 47–52.

(44) Kitoh-Nishioka, H.; Yokogawa, D.; Irle, S. Förster Resonance Energy Transfer between Fluorescent Proteins: Efficient Transition Charge-based Study. *J. Phys. Chem. C* **2017**, *121*, 4220–4238.

(45) Sarngadharan, P.; Maity, S.; Kleinekathöfer, U. Spectral Densities and Absorption Spectra of the Core Antenna Complex CP43 from Photosystem II. *J. Chem. Phys.* **2022**, *156*, 215101.

(46) Maity, S.; Kleinekathöfer, U. Recent Progress in Atomistic Modeling of Light-Harvesting Complexes: A Mini Review. *Photosynth. Res.* **2023**, *156*, 147–162.

(47) Cupellini, L.; Lipparini, F.; Cao, J. Absorption And Circular Dichroism Spectra Of Molecular Aggregates With The Full Cumulant Expansion. *J. Phys. Chem. B* **2020**, *124*, 8610–8617.

(48) Novoderezhkin, V. I.; Palacios, M. A.; van Amerongen, H.; van Grondelle, R. Energy-Transfer Dynamics in the LHCII Complex of Higher Plants: Modified Redfield Approach. *J. Phys. Chem. B* **2004**, *108*, 10363.

(49) Wang, W.; Yu, L.-J.; Xu, C.; Tomizaki, T.; Zhao, S.; Umena, Y.; Chen, X.; Qin, X.; Xin, Y.; Suga, M.; Han, G.; Kuang, T.; Shen, J.-R. Structural Basis for Blue-Green Light Harvesting and Energy Dissipation in Diatoms. *Science* **2019**, *363*.
